# Does the institutionalization influence elderly’s quality of life? A systematic review and meta–analysis

**DOI:** 10.1186/s12877-020-1452-0

**Published:** 2020-02-05

**Authors:** Mariana Marinho Davino de Medeiros, Talita Malini Carletti, Marcela Baraúna Magno, Lucianne Cople Maia, Yuri Wanderley Cavalcanti, Renata Cunha Matheus Rodrigues-Garcia

**Affiliations:** 1 Department of Prosthodontics and Periodontology, Piracicaba Dental School, University of Campinas, Piracicaba, São Paulo Brazil; 2 Department of Paediatric Dentistry and Orthodontics, Federal University of Rio de Janeiro, Rio de Janeiro, Brazil; 3 Department of Clinical and Social Dentistry, Federal University of Paraíba, João Pessoa, Paraíba Brazil

**Keywords:** Aged, Institutionalization, Nursing homes, Independent living, Quality of life

## Abstract

**Background:**

Institutionalization is a global phenomenon and its impact on elderly’s quality of life (QoL) is under discussion. This systematic review and meta-analysis evaluated the influence of the institutionalization on elderly’s QoL.

**Methods:**

Searches were performed in Medline, Scopus, Web of Science, Lilacs, Cochrane Library and SIGLE by two independent reviewers up to May 2019. The eligibility criteria were based on PECO strategy, considering observational studies in elderly (P), which were (E) or not (C) institutionalized to identify differences in their QoL (O). For qualitative synthesis, data were extracted and risk of bias was evaluated through a validated guideline. Meta-analysis was based on Mean Difference (MD) and Standard Mean Difference (SMD) calculation (*p* ≤ 0.05). The evidence was quality-tested using Grading of Recommendations Assessment, Development and Evaluation (GRADE) approach.

**Results:**

The initial search identified 3841 articles. Duplicates were removed, titles and abstracts were read and eligibility criteria were applied, remaining 16 sixteen cross-sectional studies that were included for data extraction and qualitative synthesis. Out of 16 articles, 14 evaluated the Health-Related Quality of Life, using Leipad (*n* = 2), WHOQOL-BREF and/or OLD (*n* = 8), SF-36 or RAND-36 (*n* = 4) questionnaires, and two assessed the Oral Health–Related Quality of Life, through GOHAI questionnaire. One eligible article was considered as low risk of bias. In the meta-analysis, 12 studies were included. Leipad questionnaire did not show differences on elderly’s QoL (MD 0.11 [− 0.10, 0.32] I^2^ = 76%). Differences on elderly’s QoL were detected through WHOQOL-BREF (SMD -0.70 [CI95%: − 0.94, − 0.47] I^2^ = 93%), WHOQOL-OLD (SMD -1.13 [− 1.47, − 0.80] I^2^ = 91%) and SF-36/RAND-36 (MD -5.97 [CI95%: − 11.29, − 0.64] I^2^ = 90%). All studies had very low or low certainty of evidence, since the study design influenced evidence classification, and show high heterogeneity.

**Conclusion:**

Although the institutionalization influences negatively the elderly’s QoL, further well-designed studies are needed to confirm this evidence.

## Background

The elderly population is growing worldwide in greater rates [1], as a result of increased longevity and lower mortality rates [2, 3]. In view of this, there is a concern about the active aging process, in which the continuing participation of aged people on daily activities is enhanced [4]. Active aging refers to keep elderly health and on the control of their daily activities. This may generate better Quality of Life (QoL) [5], represented by favourable perceptions of their position in life, within a cultural context, in relation to their goals, expectations, concerns and desires [6]. Therefore, active aging refers to the physical, social and mental well-being, as well as, social participation, protection, safety, and care of the elderly to avoid disabilities, chronic diseases and less use of health care services [4].

Although the importance of active aging and better QoL for the elderly is evident, there is a lack of family care, which increases the elderly’s institutionalization and, by consequence, increase the number of community-dwelling aged people who became residents of nursing homes [7]. Advance age, not having a home or a partner, low educational level, sedentary lifestyle, poor self-rated health status, high number of drug prescriptions and functional and cognitive impairments are the main predictors of the institutionalization process [7, 8]. In addition, the lack of support and assistance to the elderly during daily activities is suggested as an aggravating factor for stimulating elderly’s institutionalization [7].

Considering the raised institutionalization rates, nursing homes should provide good quality of life for their residents [9]. In this sense, studies [10–25] sought to understand if lives in homes for the aged may influence the elderly’s QoL. Thereby, worse QoL was observed in elderly residents of long-term care institutions, in comparison with community-dwelling individuals [11, 13, 16–19, 22, 23, 25]. In addition, the literature has shown that the elderly residing in nursing homes or institutionalized elderly have lower educational level [13, 25], poorer health status [13, 25], higher dependency level [18], higher risk of falls [18], lower physical activity [18, 22], lower decision-making ability [23], lower leisure activities [25] and are older [13, 25].

In contrast, a study found better QoL of institutionalized elderly men compared to the non-institutionalized elderly in physical and psychological domains [15], which was attributed to the multidisciplinary professional team offering support and stimulus to the institutionalized elderly. Other studies did not find differences in the QoL between institutionalized elderly and non-institutionalized elderly [14, 20]. Finally, divergent results, from different QoL questionnaire domains were observed between institutionalized and non-institutionalized elderly [10, 12, 21, 24].

Considering this, it is important solve such controversies in order to know if the institutionalization influence the QoL and in which domains. Thus, this knowledge can support the homes for the aged in performing actions and better care for the elderly in view of the promotion of a good QoL for these individuals. Therefore, the purpose of this systematic review and meta-analysis was to summarize these findings and verify the influence of institutionalization on the elderly’s general health and oral health related QoL.

## Methods

### Study design, focused question, registration and protocol

A systematic review and meta-analysis were conducted in order to answer the focused question: Does institutionalization interfere with elderly’s quality of life? The focused question was based on Population, Exposure, Comparison and Outcome (PECO) strategy [26]. This systematic review and meta-analysis investigated if elderly (P) who are institutionalized (E), compared to non-institutionalized (community-dwelling) (C), present worse QoL (O). Thereafter, this review was registered in the PROSPERO database (protocol number: CRD42018106641) and was performed according to the Preferred Reporting Items for Systematic Reviews and Meta-Analysis (PRISMA) [27].

### Literature search strategy

The literature search strategy was performed independently by two examiners, MMDM and TMC, up to May 2019 in the following electronic databases: PubMed (MEDLINE), Scopus, Web of Science, LILACS, Cochrane Library and System for Information on Gray Literature in Europe (SIGLE). MeSH terms, key words and free terms related to the topic of this systematic review were used within the search strategy. Boolean operators (OR, AND) was used to combine the search terms. In addition, the search strategy followed the syntax rules of each database, as shown in Table 1. Studies that covered the focused question: “Does the elderly who lives in nursing homes, compared to community-dwelling elderly, present worse QoL?”, and published up to May 2019 were included, without restriction of publication date or language. Furthermore, the references of all the selected studies were hand searched to retrieve articles that might have been lost in the search strategy. Finally, the ongoing or in press articles were searched through the contact with the experts by email and in abstracts and presentation from national and international dental meetings [28].

**Table 1:** Search strategy according to different databases

Database	Search Strategy
PubMed	#1 (((((((((((((((aged [MeSH Terms]) OR aged [Title/Abstract]) OR elderly [Title/Abstract]) OR ((Aged, 80 and over [MeSH Terms]))) OR ((“Aged, 80[Title/Abstract] AND over”[Title/Abstract]))) OR “oldest old”[Title/Abstract]) OR Nonagenarian*[Title/Abstract]) OR Octogenarian*[Title/Abstract]) OR Centenarian*[Title/Abstract]) OR “Old people”[Title/Abstract]) OR “Old person”) OR “Elders”) OR “Elderly people”) OR “Elderly person”) OR “Elderly population”) OR Seniors [Title/Abstract] #2 (((((((((((((((((((((((Institutionalization [MeSH Terms]) OR Institutionalized Person*[Title/Abstract]) OR “Person, Institutionalized”[Title/Abstract]) OR Institutionalization*[Title/Abstract]) OR Homes for the Aged [MeSH Terms]) OR “Home, Old Age”[Title/Abstract]) OR “Homes, Old Age”[Title/Abstract]) OR Old Age Home*[Title/Abstract]) OR “Geriatric Long-Term Care Facilities”[Title/Abstract]) OR “Geriatric Long-Term Care Institutions”[Title/Abstract]) OR “Homes for the Aged”[Title/Abstract]) OR Almshouses [MeSH Terms]) OR Almshouse*[Title/Abstract]) OR Poorhouse*[Title/Abstract]) OR Nursing Homes [MeSH Terms]) OR “Homes, Nursing”[Title/Abstract]) OR “Home, Nursing”[Title/Abstract]) OR Nursing Home*[Title/Abstract]) OR Housing for the elderly [MeSH Terms]) OR “Life Care Centers, Retirement”[Title/Abstract]) OR “Continuing Care Retirement Centers”[Title/Abstract]) OR “Housing for the elderly”[Title/Abstract]) OR “Institutionalized older adults”[Title/Abstract]) OR “Institutionalized elderly”[Title/Abstract] #3 (((((((((((((((Independent living [MeSH Terms]) OR “Living, Independent”[Title/Abstract]) OR “Community Dwelling”[Title/Abstract]) OR “Dwelling, Community”[Title/Abstract]) OR “Dwellings, Community”[Title/Abstract]) OR “Aging in Place”[Title/Abstract]) OR “Independent living”[Title/Abstract]) OR Deinstitutionalization [MeSH Terms]) OR “Deinstitutionalized Persons”[Title/Abstract]) OR “Deinstitutionalized Person”[Title/Abstract]) OR “Persons, Deinstitutionalized”[Title/Abstract]) OR Deinstitutionalization [Title/Abstract]) OR “Non-institutionalized elderly”[Title/Abstract]) OR “Non-institutionalized elders”[Title/Abstract]) OR Non-institutional [Title/Abstract]) OR Community [Title/Abstract] #4 ((((Quality of Life [MeSH Terms]) OR “Life Quality”[Title/Abstract]) OR “Health Related Quality Of Life”[Title/Abstract]) OR HRQOL [Title/Abstract]) OR “Quality of life”[Title/Abstract] #1 AND #2 AND #3 AND #4
Scopus	#1 TITLE-ABS-KEY (aged) OR TITLE-ABS-KEY (elderly) OR TITLE-ABS-KEY(“Oldest Old”) OR TITLE-ABS-KEY (Nonagenarian*) OR TITLE-ABS-KEY (Octogenarian*) OR TITLE-ABS-KEY (Centenarian*) OR TITLE-ABS-KEY(“aged, 80 over”) OR TITLE-ABS-KEY(“Old people”) OR TITLE-ABS-KEY(“old person”) OR TITLE-ABS-KEY (elders) OR TITLE-ABS-KEY(“elderly people”) OR TITLE-ABS-KEY(“elderly person”) OR TITLE-ABS-KEY(“elderly population”) OR TITLE-ABS-KEY (seniors) #2 TITLE-ABS-KEY (Institutionalized AND Person*) OR TITLE-ABS-KEY(“Person, Institutionalized”) OR TITLE-ABS-KEY (Institutionalization*) OR TITLE-ABS-KEY(“Home, Old Age”) OR TITLE-ABS-KEY(“Homes, Old Age”) OR TITLE-ABS-KEY (Old AND Age AND Home*) OR TITLE-ABS-KEY(“Geriatric Long-Term Care Facilities”) OR TITLE-ABS-KEY(“Geriatric Long-Term Care Institutions”) OR TITLE-ABS-KEY(“Homes for the Aged”) OR TITLE-ABS-KEY (Almshouse*) OR TITLE-ABS-KEY (Poorhouse*) OR TITLE-ABS-KEY (Nursing AND Home*) OR TITLE-ABS-KEY(“Home, Nursing”) OR TITLE-ABS-KEY(“Homes, Nursing”) OR TITLE-ABS-KEY(“Housing for the elderly”) OR TITLE-ABS-KEY(“Life Care Centers, Retirement”) OR TITLE-ABS-KEY(“Continuing Care Retirement Centers”) OR TITLE-ABS-KEY(“Institutionalized older adults”) OR TITLE-ABS-KEY(“Institutionalized elderly”) #3 TITLE-ABS-KEY(“Independent living”) OR TITLE-ABS-KEY(“Community Dwelling”) OR TITLE-ABS-KEY(“Dwelling, Community”) OR TITLE-ABS-KEY(“Dwellings, Community”) OR TITLE-ABS-KEY(“Aging in Place”) OR TITLE-ABS-KEY (Deinstitutionalization) OR TITLE-ABS-KEY(“Deinstitutionalized Persons”) OR TITLE-ABS-KEY(“Deinstitutionalized Person”) OR TITLE-ABS-KEY(“Persons, Deinstitutionalized”) OR TITLE-ABS-KEY(“Independent living”) OR TITLE-ABS-KEY(“Non-institutionalized elderly”) OR TITLE-ABS-KEY(“Non-institutionalized elders”) OR TITLE-ABS-KEY (non-institutional) OR TITLE-ABS-KEY (community) #4 TITLE-ABS-KEY(“Quality of Life”) OR TITLE-ABS-KEY(“Life Quality”) OR TITLE-ABS-KEY(“Health Related Quality of Life”) OR TITLE-ABS-KEY (HRQOL) #1 AND #2 AND #3 AND #4
Web of Science	#1 TS = (aged OR elderly OR “Oldest Old” OR Nonagenarian* OR Octogenarian* OR Centenarian* OR “Aged, 80 and over” OR “Old people” OR “old person” OR elders OR “elderly people” OR “elderly person” OR “elderly population” OR seniors) #2 TS = (Institutionalized Person* OR “Person, Institutionalized” OR Institutionalization OR Institutionalization* OR “Home, Old Age” OR “Homes, Old Age” OR Old Age Home* OR “Geriatric Long-Term Care Facilities” OR “Geriatric Long-Term Care Institutions” OR “Homes for the Aged” OR “Homes for the Aged” OR Almshouses OR Almshouse* OR Poorhouse* OR “Nursing Homes” OR Nursing Home* OR “Home, Nursing” OR “Homes, Nursing” OR “Housing for the elderly” OR “Life Care Centers, Retirement” OR “Continuing Care Retirement Centers” OR “Institutionalized older adults” OR “Institutionalized elderly”) #3 TS = (“Independent living” OR “Living, Independent” OR “Community Dwelling” OR “Dwelling, Community” OR “Dwellings, Community” OR “Aging in Place” OR Deinstitutionalization OR “Deinstitutionalized Persons” OR “Deinstitutionalized Person” OR “Persons, Deinstitutionalized” OR Deinstitutionalization OR “Independent living” OR “Non-institutionalized elderly” OR “Non-institutionalized elders” OR “non-institutional” OR community) #4 TS = (“Quality of Life” OR “Life Quality” OR “Health Related Quality Of Life” OR HRQOL) #1 AND #2 AND #3 AND #4
Cochrane Library	#1 MeSH descriptor: [Aged] explode all trees 1640 #2 aged OR elderly OR “Oldest Old” OR Nonagenarian* OR Octogenarian* OR Centenarian* 430,102 #3 MeSH descriptor: [Aged, 80 and over] explode all trees 262 #4 “Aged, 80 and over” OR “Old people” OR “old person” OR elders OR “elderly people” OR “elderly person” OR “elderly population” OR seniors 52,836 #5 #1 OR #2 OR #3 OR #4 430,609 #6 MeSH descriptor: [Institutionalization] explode all trees 200 #7 Institutionalization* OR Institutionalized Person* OR “Person, Institutionalized” 794 #8 MeSH descriptor: [Homes for the Aged] explode all trees 556 #9 “Homes for the Aged” OR “Home, Old Age” OR “Homes, Old Age”OR Old Age Home* OR “Geriatric Long-Term Care Facilities”OR “Geriatric Long-Term Care Institutions” 0 #10 MeSH descriptor: [Almshouses] explode all trees 0 #11 Almshouse* OR Poorhouse* 2 #12 MeSH descriptor: [Nursing Homes] explode all trees 1189 #13 Nursing Home* OR “Home, Nursing” OR “Homes, Nursing” 6599 #14 MeSH descriptor: [Housing for the Elderly] explode all trees 35 #15 “Housing for the elderly” OR “Care Centers, Retirement” OR “Continuing Care Retirement Centers” OR “Institutionalized older adults” OR “Institutionalized elderly” 352 #16 #6 OR #7 OR #8 OR #9 OR #10 OR #11 OR #12 OR #13 OR #14 OR #15 1,302,402 #17 MeSH descriptor: [Independent Living] explode all trees 267 #18 “Independent living” OR “Living, Independent” OR “Community Dwelling” OR “Dwelling, Community” OR “Dwellings, Community” OR “Aging in Place” 3417 #19 MeSH descriptor: [Deinstitutionalization] explode all trees 22 #20 Deinstitutionalization OR “Deinstitutionalized Persons” OR “Deinstitutionalized Person” OR “Persons, Deinstitutionalized” OR “Non-institutionalized elderly” OR “Non-institutionalized elders” OR “non-institutional” OR community 37,791 #21 MeSH descriptor: [Quality of Life] explode all trees 20,225 #22 “Quality of Life” OR “Life Quality” OR “Health Related Quality Of Life” OR HRQOL 72,184 #23 #17 OR #18 OR #19 OR #20 38,098 #24 #21 OR #22 72,184 #25 #5 AND #16 AND #23 AND #24 3450
Lilacs	#1 (mh:(aged)) OR (tw:(aged)) OR (tw:(elderly)) OR (tw:(“Oldest Old”)) OR (tw:(Nonagenarian$)) OR (tw:(Octogenarian$)) OR (tw:(Centenarian$)) OR (mh:(“Aged, 80 and over”)) OR (tw:(“Aged, 80 and over”)) OR (tw:(“Old people”)) OR (tw:(“old person”)) OR (tw:(elders)) OR (tw:(“elderly people”)) OR (tw:(“elderly person”)) OR (tw:(“elderly population”)) OR (tw:(seniors)) #2 (tw:(Institutionalized Person$)) OR (tw:(“Person, Institutionalized”)) OR (mh:(“Institutionalization”)) OR (tw:(Institutionalization$)) OR (tw:(“Home, Old Age”)) OR (tw:(“Homes, Old Age”)) OR (tw:(Old Age Home$)) OR (tw:(“Geriatric Long-Term Care Facilities”)) OR (tw:(“Geriatric Long-Term Care Institutions”)) OR (tw:(“Homes for the Aged”)) OR (tw:(“Homes for the Aged”)) OR (mh:(“Almshouses”)) OR (tw:(Almshouse$)) OR (tw:(Poorhouse$)) OR (mh:(“Nursing Homes”)) OR (tw:(Nursing Home$)) OR (tw:(“Home, Nursing”)) OR (tw:(“Homes, Nursing”)) OR (mh:(“Housing for the elderly”)) OR (tw:(“Housing for the elderly”)) OR (tw:(“Life Care Centers, Retirement”)) OR (tw:(“Continuing Care Retirement Centers”)) OR (tw:(“Institutionalized older adults”)) OR (tw:(“Institutionalized elderly”)) #3 (mh:(Independent living)) OR (tw:(“Independent living”)) OR (tw:(“Living, Independent”)) OR (tw:(“Community Dwelling”)) OR (tw:(“Dwelling, Community”)) OR (tw:(“Dwellings, Community”)) OR (tw:(“Aging in Place”)) OR (mh:(Deinstitutionalization)) OR (tw:(Deinstitutionalization)) OR (tw:(“Deinstitutionalized Persons”)) OR (tw:(“Deinstitutionalized Person”)) OR (tw:(“Persons, Deinstitutionalized”)) OR (mh:(Deinstitutionalization)) OR (tw:(Deinstitutionalization)) OR (mh:(“Non-institutionalized elderly”)) OR (tw:(“Non-institutionalized elders”)) OR (tw:(“non-institutional”)) OR (tw:(community)) #4 (mh:(“Quality of Life”)) OR (tw:(“Quality of Life”)) OR (tw:(“Life Quality”)) OR (tw:(“Health Related Quality Of Life”)) OR (tw:(HRQOL)) #1 AND #2 AND #3 AND #4
Open Grey	#1 (aged OR elderly OR “Oldest Old” OR Nonagenarian* OR Octogenarian* OR Centenarian* OR “Aged, 80 and over” OR “Old people” OR “old person” OR elders OR “elderly people” OR “elderly person” OR “elderly population” OR seniors) #2 (Institutionalized Person* OR “Person, Institutionalized” OR Institutionalization OR Institutionalization* OR “Home, Old Age” OR “Homes, Old Age” OR Old Age Home* OR “Geriatric Long-Term Care Facilities” OR “Geriatric Long-Term Care Institutions” OR “Homes for the Aged” OR “Homes for the Aged” OR Almshouses OR Almshouse* OR Poorhouse* OR “Nursing Homes” OR Nursing Home* OR “Home, Nursing” OR “Homes, Nursing” OR “Housing for the elderly” OR “Life Care Centers, Retirement” OR “Continuing Care Retirement Centers” OR “Institutionalized older adults” OR “Institutionalized elderly”) #3 (“Independent living” OR “Living, Independent” OR “Community Dwelling” OR “Dwelling, Community” OR “Dwellings, Community” OR “Aging in Place” OR Deinstitutionalization OR “Deinstitutionalized Persons” OR “Deinstitutionalized Person” OR “Persons, Deinstitutionalized” OR Deinstitutionalization OR “Independent living” OR “Non-institutionalized elderly” OR “Non-institutionalized elders” OR “non-institutional” OR community) #4 (“Quality of Life” OR “Life Quality” OR “Health Related Quality Of Life” OR HRQOL) #1 AND #2 AND #3 AND #4

### Eligibility criteria

The inclusion criteria were based on the elements of the PECO strategy [26], considering observational studies that compared elderly (P), which were (E) institutionalized or not (C), in order to identify differences in their Quality of Life (O). People aged 60 years old or more was considered elderly, following the World Health Organization (WHO) and United Nations definition. Moreover, aged people who lived in a nursing home was considered institutionalized.

References from database searches were imported into the Mendeley Desktop software (Mendeley Desktop, version 1.16.1,©2008–2016 Mendeley Ltd., Elsevier Inc., NY, USA). This reference manager software was used to remove the duplicates, followed by title and abstract reading. Two examiners (MMDM and TMC) analyzed independently the study designs and excluded references that did not meet the inclusion criteria (observational studies), such as editorials, letters to editor, literature reviews, case reports, case series. In addition, following the eligibility criteria, observational studies that did not include a group of comparison (non-institutionalized individuals) also were not included. Subsequently, titles and abstracts of the searched papers were analyzed for possible inclusion, according to the eligibility criteria. In case of title and abstract provided insufficient information to accomplish a proper exclusion, full-text was also read to resolute any doubts and the final decision was made.

In this stage, studies that met the eligibility criteria, however, were about Alzheimer’s disease, dementia, mental retardation or disability, articles that used Likert scale and Visual Analogue Scale (VAS) to assess QoL, as well as, validation studies of the QoL questionnaire were excluded of this systematic review, being these the exclusion criteria. After that, the full texts were read and evaluated. Thus, the minimum sample size considered to the studies was 61 individuals. The results of both reviewers were compared, and any inconsistency was solved with a third examiner (YWC).

### Data extraction

Data were extracted independently by the two examiners (MMDM and TMC) and organized in an electronic spreadsheet (Table 2): (1) author, year of publication and geographical location; (2) study design; (3) sample size: numbers of participants; (4) sample characteristics: gender and age; (5) data collection; and (6) results. The spreadsheets of the two examiners were compared, and if any inconsistency was founded, a third examiner (YWC) solved the doubts.

**Table 2:** Data collection of the eligible articles

Authors, year (local)	Study Design	Sample size	Sample characteristics	Data collection	Results
Urciuoli et al., 1998 [24] (Italy)	Cross-sectional	Convenience sample (n = 66, being 29 IE and 37 NIE)	IE = 4 male and 25 females; NIE = 6 male and 31 females Age: > 88 years	LEIPAD (The lower the scores, the better the QoL) Scale: 0–93 points	Physical functions: IE: mean = 7.20, SD = 1.91; NIE: mean = 6.55, SD = 2.37 (*p* > 0.05, Student’s T-test) Self-care skills: IE: mean = 12.86, SD = 3.39; NIE: mean = 10.45, SD = 5.58 (*p* < 0.05, Student’s T-test) Cognitive functions: IE: mean = 4.50, SD = 2.08; NIE: mean = 4.74, SD = 2.91 (*p* > 0.05, Student’s T-test) Depression and anxiety: IE: mean = 1.79, SD = 2.42; NIE: mean = 2.23, SD = 2.17 (*p* > 0.05, Student’s T-test) Social functions: IE mean = 3.48, SD = 1.80; NIE: mean = 2.79, SD = 1.93 (*p* > 0.05, Student’s T-test) Sexual functions: IE mean = 6.00, SD = 0.00; NIE: mean = 6.00, SD = 0.00 (*p* > 0.05, Student’s t test) Life Satisfaction: IE mean = 4.75, SD = 2.31; NIE: mean = 5.52, SD = 2.83 (p > 0,05, Student’s T-test)
Akça; Sahin, 2008 [10] (Turkey)	Cross-sectional	Convenience sample (*n* = 251, being 90 IE and 124 NIE)	IE = 52 male and 38 females; NIE = 87 male and 37 females Age: ≥ 60 years	LEIPAD (The lower the scores, the better the QoL) Scale: 0–93 points	Physical functions: IE mean = 12.70, SD = 2.67; NIE: mean = 12.46, SD = 2.51 (*p* > 0.05, Student’s T-test) Self-care skills: IE mean = 9.63, SD = 4.76; NIE: mean = 10.28, SD = 5.00 (*p* > 0.05, Student’s T-test) Cognitive functions: IE mean = 11.28, SD = 2.39; NIE: mean = 12.04, SD = 2.43 (*p* < 0.05, Student’s T-test) Depression and anxiety: IE mean = 9.14, SD = 2.95; NIE: mean = 9.94, SD = 3.29 (*p* > 0.05, Student’s T-test) Social functions: IE mean = 7.94, SD = 1.91; NIE: mean = 7.21, SD = 2.28 (*p* < 0.05, Student’s T-test) Sexual functions: IE mean = 7.43, SD = 1.25; NIE: mean = 6.33, SD = 1.50 (*p* < 0.05, Student’s T-test) Life Satisfaction: IE mean = 17.19, SD = 2.66; NIE: mean = 16.90, SD = 3.20 (*p* > 0.05, Student’s T-test)
Bonan et al., 2008 [14] (Brazil)	Cross-sectional	Convenience Sample (*n* = 90, being 45 IE and 45 NIE)	IE = 22 male and 23 females; NIE = 20 male and 25 females Age: > 55 years	GOHAI (The higher the scores, the better the QoL) Scale: 0–60 points	IE: mean = 50, SD = 8; NIE: mean = 50, SD = 6 (*p* > 0.05, Student’s T-test)
Bodur; Cingil, 2009 [13] (Turkey)	Cross-sectional	Convenience Sample (*n* = 74, being 37 IE and 37 NIE)	IE = 60% male and 40% females; NIE = 38% male and 62% females Age: > 60 years	WHOQOL-BREF (The higher the scores, the better the QoL) Scale: 0–100 points	General Health: IE: mean = 71.9, SD = 20.4; NIE: mean = 65.1, SD = 18.8 (*p* > 0.05, Student’s T-test) Physical Health: IE: mean = 62.2, SD = 29.7; NIE: mean = 58.1, SD = 22.3 (*p* > 0.05, Student’s T-test) Psychological Health: IE: mea*n* = 66.2, SD = 24.7; NIE: mean = 63.1, SD = 20.3 (*p* > 0.05, Student’s T-test) Social relationship: IE: mean = 58.1, SD = 23.7; NIE: mean = 73.9, SD = 23.0 (*p* < 0.05, Student’s T-test) Environmental area: IE: mean = 66.5, SD = 17.2; NIE: mean = 75.9, SD = 17.9 (*p* < 0.05, Student’s T-test)
Alcarde et al., 2010 [11] (Brazil)	Cross-sectional	Convenience Sample (*n* = 137, being 90 IE and 47 NIE)	IE = 68 male and 69 females; NIE = 68 male and 69 females Age: 60 to 92 years	GOHAI (The higher the scores, the better the QoL) Scale: 0–60 points	IE: median ≤ 28 (*n* = 55, 61.1%) and > 28 (*n* = 35, 38.9%); NIE: median ≤ 28 (*n* = 18, 38.3%) and > 28 (*n* = 29, 61.7%) (*p* < 0,05, Chi-square test)
Bodner et al., 2011 [12] (Israel)	Cross-sectional	Convenience Sample (*n* = 126, being 32 IE and 94 NIE)	IE = 33,4% male and 65,6% females; NIE = 47,8% male and 52,2% females Age: > 64 years	SF-36 (The higher the scores, the better the QoL) Scale: 0–100 points	The ‘general health perceptions’, ‘physical functioning’, ‘physical role functioning’, ‘bodily pain’, ‘vitality’ and ‘emotional role functioning’ did not show significant difference between the IE and NIE Mental Health: IE: mean = 51.23, SD = 29.82; NIE: mean = 79.45, SD = 12.78 (*p* < 0.05, MANCOVA) Social Functioning: IE: mean = 94.94, SD = 22.54; NIE: mean = 76.70, SD = 20.09 (*p* < 0.05, MANCOVA)
Ramos et al., 2012 [23] (South Africa)	Cross-sectional	Convenience sample (*n* = 284, being 73 IE and 175 NIE)	Distribution according sex not informed Age: > 60 years	WHOQOL-OLD (The higher the scores, the better the QoL) Scale: 0–100 points	Sensorial abilities: IE: mean = 40, SD = 15.1; NIE: mean = 50.2, SD = 14.2 (*p* < 0.05, Student’s t test) Autonomy: IE: mean = 28.3, SD = 16.5; NIE: mean = 36.5, SD = 20.9 (*p* > 0.05, Student’s t test) Past, present and future activities: IE: mean = 38.5, SD = 15.2; NIE: mean = 52.6, SD = 13.4 (*p* < 0.05, Student’s t test) Death and dying: IE: mean = 61.2, SD = 15.7; NIE: mean = 72.6, SD = 15.2 (*p* < 0.05, Student’s t test) Social participation: IE: mean = 63.6, SD = 17.1; NIE: mean = 76.2, SD = 16.1 (*p* < 0.05, Student’s t test) Intimacy: IE: mean = 57.3, SD = 21.7; NIE: mean = 74.7, SD = 21.7 (*p* < 0.05, Student’s t test)
Vitorino et al., 2013 [25] (Brazil)	Cross-sectional	Convenience sample (*n* = 354, being 66 IE and 288 NIE)	IE = 38 male and 38 females; NIE = 94 male and 194 females Age: 60 to 80 or older	WHOQOL-BREF (The higher the scores, the better the QoL) Scale: 0–100 points	General Health: IE: mean = 69.83, SD = 19.18; NIE: mean = 69.81, SD = 19.01 (*p* > 0.05, Student’s t test) Physical Health: IE: mean = 63.6, SD = 22.14; NIE: mean = 68.61, SD = 18.26 (*p* > 0.05, Student’s t test) Psychological Health: IE: mean = 65.19, SD = 17.62; NIE: mean = 69.69, SD = 15.33 (*p* < 0.05, Student’s t test) Social relationship: IE: mean = 67.87, SD = 20.31; NIE: mean = 75.10, SD = 17.27 (*p* < 0.05, Student’s t test) Environmental area: IE: mean = 66.20, SD = 15.42; NIE: mean = 65.09, SD = 16.19 (*p* > 0.05, Student’s t test)
Even-Zohar, 2014 [17] (Israel)	Cross-sectional	Convenience Sample (*n* = 115, being 60 IE and 55 NIE)	IE = 20 male and 40 females; NIE = 23 male and 32 females Age: IE: mean = 74.7 and NIE: mean = 75.8	WHOQOL-BREF (The higher the scores, the better the QoL) Scale: 0–20 points	Physical health: IE: mean = 3.06, SD = 0.457; NIE: mean = 3.70, SD = 0.623. (*p* < 0.05, Multivariate analysis of covariance) Psychological health: IE: mean = 3.03, SD = 0.42; NIE: mean = 3.82, SD = 0.57 (*p* < 0.05, Multivariate analysis of covariance) Social relationship: IE: mean = 2.90, SD = 0.81; NIE: mean = 4.06, SD = 0.62 (*p* < 0.05, Multivariate analysis of covariance) Environmental area: IE: mean = 2.96, SD = 0.46; NIE mean = 3.85, SD = 0.57 (*p* < 0.05, Multivariate analysis of covariance)
Khoury; Sá-Neves, 2014 [19] (Brazil)	Cross-sectional	Convenience Sample (n = 66, being 33 IE and 33 NIE)	IE = 13 male and 20 females; NIE = 8 male and 25 females Age: 60 to 96 years	WHOQOL-OLD (The higher the scores, the better the QoL) Scale: 0–100 points	Sensory Abilities: IE: mean = 27.86; NIE: mean = 39.14 (*p* < 0.05, Mann-Whitney test) Autonomy: IE: mean = 26.76; NIE: mean = 40.24 (*p* < 0.05, Mann-Whitney test) Past, present and future activities: IE: mean = 27.95; NIE: mean = 39,05 (*p* < 0.05, Mann-Whitney test) Death and dying: IE: mean = 33.17; NIE mean = 33.83 (*p* > 0.05, Mann-Whitney test) Social participation: IE: mean = 28.17; NIE: mean = 38.83 (*p* < 0.05, Mann-Whitney test) Intimicy: IE: mean = 27.48; NIE: mean = 39.52 (*p* < 0.05, Mann-Whitney test)
Dagios et al., 2015 [16] (Brazil)	Cross-sectional	Convenience Sample (*n* = 136, being 36 IE and 100 NIE)	IE = 25 male and 11 females; NIE = 37 male and 63 females Age: > 60 years	WHOQOL-BREF and WHOQOL-OLD (The higher the scores, the better the QoL) Scale: 0–20 points	WHOQOL-BREF General Health: IE: mean = 10.17, SD = 3.04; NIE: mean = 14.06, SD = 3.84 (*p* < 0.05, Student’s T-test) Physical Health: IE: mean = 10.08, SD = 3.32; NIE: mean = 14.61, SD = 2.73 (*p* < 0.05, Student’s T-test) Psychological Health: IE: mean = 11.35, SD = 2.65; NIE: mean = 16.02, SD = 2.54 (*p* < 0.05, Student’s T-test) Social relationship: IE: mean = 10.67, SD = 3.12; NIE: mean = 15.28, SD = 2.66 (*p* < 0.05, Student’s T-test) Environmental area: IE: mean = 10.64, SD = 1.73; NIE: mean = 12.88, SD = 2.08 (*p* < 0.05, Student’s T-test) WHOQOL-OLD Sensory Abilities: IE: mean = 11.00, SD = 3.06; NIE: mean = 15.69, SD = 3.26 (*p* < 0.05, Student’s T-test) Autonomy: IE: mean = 9.21, SD = 2.53; NIE: mean = 14.69, SD = 2.04 (*p* < 0.05, Student’s T-test) Past, Present and Future Activities: IE: mean = 9.64, SD = 3.0; NIE: mean = 15.12, SD = 2.65 (*p* < 0.05, Student’s T-test) Death and dying: IE: mean = 14.21, SD = 3.14; NIE: mean = 14.69, SD = 3.07 (*p* > 0.05, Student’s T-test) Social Participation: IE: mean = 9.30, SD = 3.64; NIE: mean = 14.93, SD = 2.80 (*p* < 0.05, Student’s T-test) Intimicy: IE: mean = 10.22, SD = 2.85; NIE: mean = 15.52, SD = 3.59 (*p* < 0.05, Student’s T-test)
Rachadel et al., 2015 [21] (Brazil)	Cross-sectional	Convenience sample (*n* = 61, being 21 IE and 40 NIE)	Distribution according sex not informed Age: > 60 years	SF-36 (The higher the scores, the better the QoL) Scale: 0–100 points	Physical functioning: IE: mean = 35.9, SD = 25.9; NIE-Active: mean = 78, SD = 19.6; NIE-Not-Active: mean = 51, SD = 27.3 (*p* < 0.05, Kruskal-Wallis) Role physical: IE: mean = 79.7, SD = 33.1; NIE-Active: mean = 62.5, SD = 39.3; NIE-Not-Active: mean = 52.5, SD = 41.2 (*p* > 0.05, Kruskal-Wallis) Bodily pain: IE: mean = 83.2, SD = 21.6; NIE-Active: mean = 61.4, SD = 25.9; NIE-Not-Active: mean = 54.4, SD = 33.3 (*p* < 0.05, Kruskal-Wallis) General Health Perceptions: IE: mean = 68.1, SD = 20.5; NIE-Active: mean = 68.4, SD = 22.2; NIE-Not-Active: mean = 58.7, SD = 29.9 (*p* > 0.05, Kruskal-Wallis) Role Emotional: IE: mean = 87.3, SD = 12.8; NIE -Active: mean = 73.3, SD = 35.2; NIE-Not-Active: mean = 76.6, SD = 34.3 (*p* > 0.05, Kruskal-Wallis) Vitality: IE: mean = 70.7, SD = 12.4; NIE-Active: mean = 69.7, SD = 19.7; NIE-Not-Active: mean = 66.0, SD = 23.4 (*p* > 0.05, Kruskal-Wallis) Mental health: IE: mean = 78.1, SD = 24.2; NIE -Active: mean = 77.2, SD = 19.6; NIE-Not-Active: mean = 69, SD = 27.9 (*p* > 0.05, Kruskal-Wallis) Social functioning: IE: mean = 95.8, SD = 4.4; NIE-Active: mean = 85, SD = 23.5; NIE-Not-Active: mean = 78.7, SD = 30.6 (*p* > 0.05, Kruskal-Wallis)
Cucato et al., 2016 [15] (Brazil)	Cross-sectional	Convenience Sample (*n* = 496, being 99 IE and 387 NIE)	IE = 24 male e 75 females; NIE = Living with family: 110 male and 170 females, Living alone: 42 male and 75 females Age: > 65 years	WHOQOL-BREF (The higher the scores, the better the QoL) Scale: 0–20 points	Institutionalized elderly men presented higher scores in physical domains compared to non-institutionalized elderly men that lives alone (*p* < 0.05, ANOVA). The scores in all domains (physical, psychological, relationship, and environment) were similar among the three groups (*p* < 0.05, ANOVA)
Herazo-Beltrán et al., 2017 [18] (Colombia)	Cross-sectional	Convenience Sample (*n* = 245, being 113 IE and 132 NIE)	IE = 48 male and 65 females; NIE = 56 male and 75 females Age: Not informed	SF-36 (The higher the scores, the better the QoL) Scale: 0–100 points	Physical Functioning: IE mean = 49.5, SD = 30.4; NIE: mean = 75.4, SD = 25.6 (*p* < 0.05, Student’s T-test) Role physical: IE mean = 35.8, SD = 40.6; NIE: mean = 57.2, SD = 40.5 (*p* < 0.05, Student’s T-test) Bodily Pain: IE mean = 62.5, SD = 30.1; NIE: mean = 69.1, SD = 27.3 (*p* > 0.05, Student’s T-test) General Health Perceptions: IE mean = 58.2, SD = 21.3; NIE: mean = 59.5, SD = 18.6 (*p* > 0.05, Student’s T-test) Role Emotional: IE mean = 45.1, SD = 43.1; NIE: mean = 61.6, SD = 43.4 (*p* < 0.05, Student’s T-test) Vitality: IE mean = 64.1, SD = 23.8; NIE: mean = 68.1, SD = 19.2 (*p* > 0.05, Student’s T-test) Mental Health: IE mean = 64.8, SD = 22.8; NIE: mean = 68.6, SD = 24.5 (*p* > 0.05, Student’s T-test) Social Functioning: IE mean = 69.9, SD = 24.8; NIE: mean = 75.7, SD = 23.1 (*p* > 0.05, Student’s T-test)
Kuok et al., 2017 [20] (China)	Cross-sectional	Randomly selected (*n* = 451, being 248 IE and 203 NIE)	IE = 35 male and 213 females; NIE = 61 male and 142 females Age: ≥50 years	WHOQOL-BREF (The higher the scores, the better the QoL) Scale: 0–20 points	Physical Health: IE: mean = 13.0, SD = 2.6; NIE: mean = 14.6, SD = 2.2 (*p* > 0.05, ANCOVA) Psychological Health: IE: mean = 13.2, SD = 2.4; NIE: mean = 14.6, SD = 2.2 (*p* > 0.05, ANCOVA) Social relationship: IE: mean = 14.0, SD = 2.6; NIE: mean = 14.4, SD = 2.3 (*p* > 0.05, ANCOVA) Environmental area: IE: mean = 13.5, SD = 2.0; NIE: mean = 13.7, SD = 2.0 (*p* > 0,05, ANCOVA)
Ramocha et al., 2017 [22] (South Africa)	Cross-sectional	Convenience sample (*n* = 80, being 40 IE and 40 NIE)	IE = 23 male and 17 females; NIE = 0 male and 40 females Age: 60 to 90 years	RAND-36 (The higher the scores, the better the QoL) Scale: 0–100 points	Physical functioning: IE: mean = 74.7, SD = 29.6; NIE: mean = 81.1, SD = 22.9 (*p* > 0.05, Student’s t test) Role physical: IE: mean = 61.2, SD = 47.3; NIE: mean = 68.1, SD = 44.2 (*p* > 0.05, Student’s t test) Bodily pain: IE: mean = 66.7, SD = 28.9; NIE: mean = 73.8, SD = 26.4 (*p* > 0.05, Student’s t test) General Health Perceptions (General Health): IE: mean = 66.1, SD = 20; NIE: mean = 73.0, SD = 18.9 (*p* > 0.05, Student’s t test) Role Emotional: IE: mean = 59.1, SD = 46.2; NIE: mean = 74.1, SD = 42.3 (*p* > 0.05, Student’s t test) Vitality (Energy and Fatigue): IE: mean = 66.3, SD = 20.5; NIE: mean = 79.5, SD = 19.1 (*p* < 0.05, Student’s t test) Mental Health (Emotional well-being): IE: mean = 73.9, SD = 19.0; NIE: mean = 86.8, SD = 13.1 (*p* < 0.05, Student’s t test) Social functioning: IE: mean = 68.9, SD = 21.4; NIE: mean = 77.1, SD = 20.5 (*p* > 0.05, Student’s t test)

Notes: *IE* Institutionalized Elderly, *NIE* Non-Institutionalized Elderly, *SD* Standard Deviation

### Quality assessment and risk of bias

Two examiners (MMDM and TMC) carried out the evaluation of the methodological quality of included studies, according to Fowkes and Fulton guidelines [29]. The guidelines proposed a checklist for appraising a medical article based in the following domains: (1) study design appropriate to objectives; (2) representativeness of study sample; (3) control group; (4) quality of measurements and outcomes; (5) completeness; and (6) distorting influences. In addition, each guideline criteria were classified according to the authors decision, after reading the content of the eligible articles, as shown in Table 3.

**Table 3:** Fowkes and Fulton criteria classification determined by the authors

Guideline	Checklist	Classification		
		0	+	++
Study sample representative?	Source of sample	Included many long-term institutions for elderly	Included a single long-term institution for elderly, but it was the unique on local	Included a single long-term institution for elderly, even with more institutions to be included
	Sampling method	Random sample	Convenience sample, but it was a cense	Convenience sample and not a cense
	Sample size	High power of study (equal or greater than 80%)	Median power of study (between 75 and 80%)	Low power of study (lower than 75%)
	Entry criteria/ exclusions	inclusion and exclusion criteria well defined, namely, presented both criteria	Inclusion and exclusion criteria not well defined, namely, presented only one of them	No criteria presented
	Non-respondents	Response rate of 100%	Response rate between 80 and 99%	Response rate lower than 80%
Control group acceptable?	Definition of controls	Well-defined control (adequate to the aim of the study)	Control group not well defined (inadequate to the aim of the study)	Control group not defined
	Source of controls	Control group from the same city of IE and/or with comparable characteristics	Control group came from different locations (non-comparable characteristics) and/or physical activities programs, elderly group, etc.	Did not mention where the control group came from
	Matching/ randomisation	Case-control relation: 1:2; 1:3, etc.	Case-control relation: 1:1	Case-control relation: 2:1; 3:1, etc.
	Comparable characteristics	Paired by age, gender, socioeconomical characteristics and comorbidity	Paired by only one of the criteria: age, gender, socioeconomical characteristics or comorbidity	Not paired
Quality of measurements and outcomes?	Validity	Used a questionnaire validated and adapted to the target language and population and/or with a good Cronbach’s alpha	Used a questionnaire validated but not adapted to the target language and population and/or with a good Cronbach’s alpha	Did not use a questionnaire validated and adapted to the target language and population and/or with a good Cronbach’s alpha
	Reproducibility	Used a validated questionnaire and performed kappa test, repeatability of measures and/or checking of measures	Used a validated questionnaire, but did not performed kappa test, repeatability of measurements and/or checking of measurements; or did not use a validated questionnaire, but did kappa test, repeatability of measurements and/ or checking of measurement	Did not used a validated questionnaire and did not perform kappa test, test and retest, etc
	Blindness	NA	NA	NA
	Quality control	Single interviewer questionnaire research	Interview questionnaire, applied by many researchers	Self-applied questionnaire
Completeness?	Compliance	NA	NA	NA
	Drop outs	NA	NA	NA
	Deaths	NA	NA	NA
	Missing data	No loss	Up to 20% of loss	More than 20% of loss
Distorting influences?	Extraneous treatments	NA	NA	NA
	Contamination	NA	NA	NA
	Changes over time	NA	NA	NA
	Confounding factors	No confounding factors	Some confounding factor (cognitive capacity or comorbidity)	Many confounding factors (cognitive capacity, comorbidity, etc)
	Distortion reduced by analysis	All confounding factors were reduced in data analysis	Some confounding factors were reduced in data analysis	Confounding factors were not reduced in data analysis

Notes: 0: No problem; +: Minor problem; ++: Major problem; *NA* Not Applicable

This classification helped to score each part of the domains of the checklist using a symbol, with the following meanings: major problem (++), minor problem (+), no problem (0) or not applicable (NA). After evaluating studies according to Fowkes and Fulton guidelines [29], the two examiners classified the studies according to the presence or absence of: (1) bias; (2) confounding factors; and (3) results occurred by chance. Studies without any problem within their domains or that solved the problems were considered sound. After quality assessment and in cases of divergence, a third researcher (YWC) proposed a consensus for the analysis.

### Meta-analysis (quantitative synthesis)

The data were analysed using RevMan software (Review Manager v. 5.3, The Cochrane Collaboration; Copenhagen, Denmark) to assess the influence of the institutionalization on the elderly’s QoL. Different questionnaires were used in the studies (LEIPAD, WHOQOL-OLD, WHOQOL-BREF and SF-36, RAND-36). Therefore, separated meta-analysis (MA) was performed for each group of QoL questionnaires [30]. Sub-grouped analysis was performed according to the domains included in each questionnaire [30]. For the MA report, the mean difference was applied to the study outcomes using the same scale range; the standard mean difference was applied to the studies with different scale ranges [31]. In all analysis, a 95% confidence interval (CI) and random effect model were applied. Heterogeneity was tested using the I^2^ index.

### Certainty of evidence

The certainty of the evidence (certainty in the estimates of effect) was determined for the outcome using the Grading of Recommendations Assessment, Development and Evaluation (GRADE) approach [32]. Observational studies start as low evidence, and the quality of the body of evidence decreases to very low if serious or very serious issues related to risk of bias, inconsistency, indirectness, imprecision and publication bias are present. In addition, the quality of the evidence can be upgraded if the magnitude of effect is large or very large, or if the effect of all plausible confounding factors would reduce the effect, or suggest a spurious effect. In this way, the quality of the evidence can vary from very low to high.

## Results

### Study selection

**Fig. 1:**
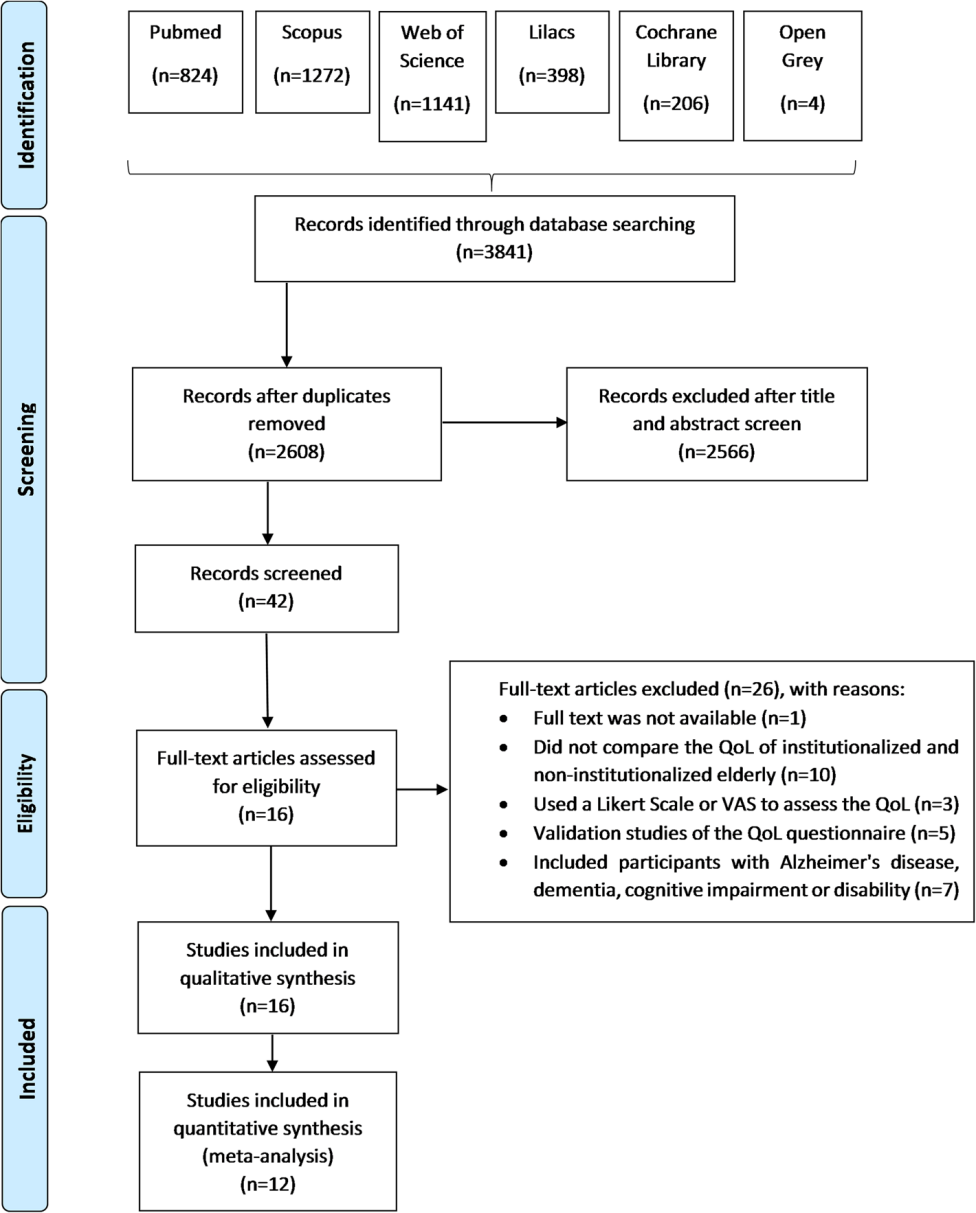
PRISMA flow diagram of literature searches

A diagram of the source and selection procedures, according to the PRISMA guidelines [27], is shown in Fig. 1. The initial search identified a total of 3841 references. Of this total, 1233 duplicates were removed, remaining 2608 studies. Title and abstract screening resulted in exclusion of 2566 records according to the eligibility criteria. Thus, 42 studies were selected for full-text reading. From that, 26 articles were excluded: one full text was not available (even after three attempts of contact with authors) and 25 did not meet the eligibility criteria. Out of these 25 studies, 10 did not compare the QoL of institutionalized and non-institutionalized elderly; three used Likert scale or VAS for QoL evaluation; five were validation studies of the QoL questionnaire; and seven included participants with Alzheimer’s disease, dementia, cognitive impairment or disability. Sixteen studies were included for the data extraction and qualitative synthesis [10–25] and 12 for the quantitative synthesis [10, 12, 13, 16–18, 20–25].

### Characteristics of included articles

Characteristics of included studies are detailed in Table 4. All retrieved papers adopted the cross-sectional design. The articles were published between 1998 [24] and 2017 [18, 20, 22], in seven different countries. Out of all included studies, seven (43,75%) were performed in Brazil. The sample sizes ranged from 61 (21 institutionalized elderly and 40 non-institutionalized elderly) [21], to 354 (66 institutionalized elderly and 288 non-institutionalized elderly) [25]. The lowest cut-off point for age considered in the studies was 50 years [20] and the highest was 88 years [24]. Furthermore, 56,25% (*n* = 9) of the studies included considered 60 years as the cut-off point for age [10, 11, 13, 16, 19, 21–23, 25].

**Table 4:** Eligible articles quality assessment, following Fowkes and Fulton guidelines

Guideline	Checklist	Urciuoli et al., 1998 [24]	Akça; Sahin, 2008 [10]	Bonan et al., 2008 [14]	Bodur; Cingil, 2009 [13]	Alcarde et al., 2010 [11]	Bodner et al., 2011 [12]	Ramos et al., 2012 [23]	Vitorino et al., 2013 [25]	Even-Zohar, 2014 [17]	Khoury; Sá-Neves, 2014 [19]	Dagios et al., 2015 [16]	Rachadel et al., 2015 [21]	Cucato et al., 2016 [15]	Herazo-Beltrán et al., 2017 [18]	Kuok et al., 2017 [20]	Ramocha et al., 2017 [22]
Study design appropriate to objectives?	Cross-sectional	X	X	X	X	X	X	X	X	X	X	X	X	X	X	X	X
	Cohort	NA	NA	NA	NA	NA	NA	NA	NA	NA	NA	NA	NA	NA	NA	NA	NA
	Controlled trial	NA	NA	NA	NA	NA	NA	NA	NA	NA	NA	NA	NA	NA	NA	NA	NA
	Case control	NA	NA	NA	NA	NA	NA	NA	NA	NA	NA	NA	NA	NA	NA	NA	NA
Study sample representative?	Source of sample	0	0	0	+	++	0	0	0	0	0	++	0	0	0	0	0
	Sampling method	++	++	++	++	++	++	++	++	++	++	++	++	++	++	+	0
	Sample size	++	0	++	0	++	0	0	++	0	++	0	0	0	0	0	++
	Entry criteria/ exclusions	+	++	+	++	++	+	+	+	++	+	+	+	+	+	+	0
	Non-respondents	0	0	0	0	0	+	0	0	0	0	++	0	0	0	+	0
Control group accetable?	Definition of controls	0	0	0	0	0	0	0	0	0	0	0	0	0	0	0	0
	Source of controls	+	+	+	+	+	0	+	+	+	+	+	+	+	+	0	+
	Matching / randomisation	+	+	+	+	++	0	0	0	+	+	0	0	0	+	+	+
	Comparable characteristics	+	++	+	+	++	+	++	++	+	++	++	++	+	+	++	+
Quality of measurements and outcomes?	Validity	0	0	0	0	0	0	0	0	0	0	0	0	0	0	0	0
	Reproducibility	+	+	+	+	+	+	+	+	+	+	+	+	+	0	+	+
	Blindness	NA	NA	NA	NA	NA	NA	NA	NA	NA	NA	NA	NA	NA	NA	NA	NA
	Quality control	+	++	+	0	+	+	+	+	+	+	0	+	0	0	0	+
Completeness?	Compliance	NA	NA	NA	NA	NA	NA	NA	NA	NA	NA	NA	NA	NA	NA	NA	NA
	Drop outs	NA	NA	NA	NA	NA	NA	NA	NA	NA	NA	NA	NA	NA	NA	NA	NA
	Deaths	NA	NA	NA	NA	NA	NA	NA	NA	NA	NA	NA	NA	NA	NA	NA	NA
	Missing data	0	0	0	0	0	+	0	0	0	0	++	0	0	0	+	0
Distorting influences?	Extraneous treatments	NA	NA	NA	NA	NA	NA	NA	NA	NA	NA	NA	NA	NA	NA	NA	NA
	Contamination	NA	NA	NA	NA	NA	NA	NA	NA	NA	NA	NA	NA	NA	NA	NA	NA
	Changes over time	NA	NA	NA	NA	NA	NA	NA	NA	NA	NA	NA	NA	NA	NA	NA	NA
	Confounding factors	+	++	+	+	++	+	++	++	+	++	++	++	+	+	+	+
	Distortion reduced by analysis	++	++	++	++	++	++	++	++	0	++	++	++	++	++	0	++
Summary questions	Bias	Yes	Yes	Yes	Yes	Yes	Yes	Yes	Yes	No	Yes	Yes	Yes	Yes	Yes	No	Yes
	Confounding	No	Yes	No	Yes	Yes	No	Yes	Yes	Yes	Yes	Yes	Yes	No	No	No	No
	Chance	Yes	Yes	Yes	No	Yes	No	Yes	Yes	No	Yes	Yes	Yes	No	No	No	Yes

Notes: 0: No problem; +: Minor problem; ++: Major problem; NA: Not Applicable

Of 16 articles evaluated in this systematic review, 14 evaluated the Health-Related Quality of Life (HRQoL) using Leipad (*n* = 2) [10, 24], WHOQOL-BREF and/or OLD (*n* = 8) [13, 15–17, 19, 20, 23, 25], SF-36 or RAND-36 (*n* = 4) [12, 18, 21, 22] questionnaires. Two studies assessed the Oral Health–Related Quality of Life (OHRQoL), through GOHAI questionnaire [11, 14].

Eight studies reported that institutionalization impacted negatively the elderly’s HRQoL [13, 16–19, 22, 23, 25]. However, one study found better HRQoL in the institutionalized elderly compared to the non-institutionalized elderly [15]. In addition, one study did not find a significant difference in the HRQoL of institutionalized elderly compared to the non-institutionalized elderly [20]. In relation to the OHRQoL, one study showed that institutionalized elderly had worse QoL compared to non-institutionalized elderly [11], whilst other paper did not find a significant difference in the QoL between the groups [14].

### Risk of bias within studies (qualitative synthesis)

The risk of bias assessment [29] is presented in Table 3. Thirteen articles (81.2%) selected the participants in more than one nursing homes [10, 12, 14, 15, 17–25], which was considered as “no problem” (0) once it provides a more representative sample of the population. Fourteen studies (87.6%) used a convenience sample as the sampling method [10–19, 21, 23–25] and was classified as “major problem” (++). The sample size was evaluated according to the power of the study that was considered high (equal to or greater than 80%) in 62.5% (*n* = 10) of the studies included in this systematic review [10, 12, 13, 15–18, 20, 21, 23]. In contrast, eleven articles (68,7%) presented only the inclusion or exclusion criteria, classified as minor problem (+) [12, 14–16, 18–21, 23, 25]. For this reason, it is possible that confounding factors exists. Despite of this, a response rate of 100% was present in thirteen studies (91.2%) [10, 11, 13–15, 17–19, 21–24].

All the articles included in this systematic review correctly defined the control group. In another hand, in relation to the source of controls, 87.5% (*n* = 14) of the articles selected the non-institutionalized elderly (control group) from physical activity programs for the aged and elderly individuals [10, 11, 13–19, 21–25]. This was considered as a “minor problem” (++) due to the control group may not have similar characteristics to the elderly from nursing homes (case group); as a result, the comparison of the characteristics of the two groups, case and control group, may be compromised. Of the articles included in the qualitative analysis, nine (56.2%) presented a ratio of 1:1 between groups, which is classified as a minor problem (+) [10, 13, 14, 17–20, 22, 24]. In addition, in relation to the topic “comparable characteristics” evaluated in the qualitative synthesis, 50.0% of the studies (*n* = 8) had major problems (++) [10, 11, 16, 19–21, 23, 25]. These articles did not match the case group (institutionalized elderly) with the control group (non-institutionalized elderly) regarding age, sex, socioeconomic characteristics and comorbidities.

Ten studies (62.5%) applied the questionnaire through an interview by more than one interviewer [11, 12, 14, 17, 19, 21–25]. Questionnaire application by means of interview is positive, considering that the participants are aged people. However, having more than one interviewer is negative, as it promotes different results, considering that these articles did not calibrate the interviewers. Therefore, this was considered a minor problem (+). Of the articles included in the qualitative synthesis, 43.75% (*n* = 7) and 56.25% (*n* = 9) had major (++) and minor problems (+), respectively, since the study had confounding factors, such as participants presenting cognitive impairment and/or comorbidities. In addition, the confounding factors and the lack of compatibility of characteristics between the groups were not reduced in data analysis of 13 articles (87.5%), being a major problem (++) [10–16, 18, 19, 21–23, 25]. Therefore, the included studies in the qualitative synthesis presented methodological problems that were considered as high risk of bias. In the end, out of 16 eligible articles, one (6.2%) was considered as low risk of bias [20].

### Meta-analysis and certainty of evidence

Of the 16 included studies, four were not included in the MA due to insuficient data [11, 14, 15, 19], remaining 12 eligible articles for the MA. The results were presented separately for MA:

### LEIPAD questionnaire

**Fig. 2:**
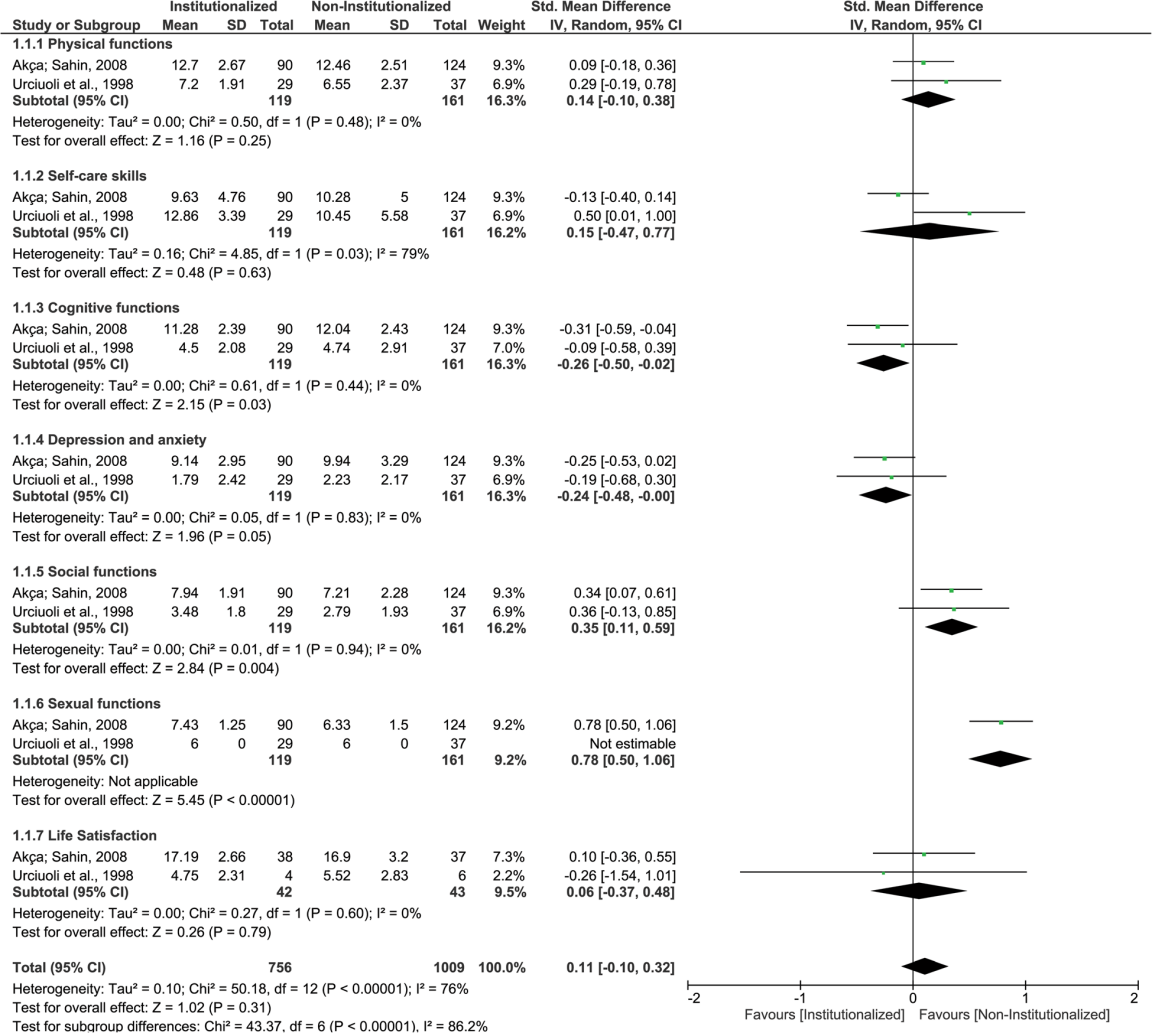
Forest plot of the influence of institutionalization on the elderly’s quality of life according to the studies that used LEIPAD questionnaire

Two studies were included in this analysis. It could be observed that institutionalized elderly presented lower mean scores (better QoL) than non-institutionalized elderly for ‘cognitive functions’ and ‘depression and axiety’ domains, while NIE presented lower mean scores (better QoL) than institutionalized elderly for ‘social functions’ and ‘sexual functions’ domains (Fig. 2). These four domains results were classified as having very low certainty of evidence. While institutionalized elderly and non- institutionalized elderly presented similar mean scores (QoL) for ‘physical functions’, ‘self-care skils’, ‘life satisfaction’ and for pooled results (Fig. 2 and Table 5), with low, very low, low and very low centainty of evidence, respectively. The GRADE classifications and reasons for each LEIPAD questionnaire domain and pooled results are described in Table 6.

**Table 5:** Numerical results according questionnaire and respective domains and polled results

Questionnaire	Questionnaire domain		*p*-value	I^2^
LEIPAD	cognitive functions	SMD −0.26 [−0.50, −0.02]	0.03	0%
	depression and axiety	SMD −0.24 [−0.48, −0.00]	0.05	0%
	social functions	SMD 0.35 [0.11, 0.59]	0.004	0%
	sexual functions	SMD 0.78 [0.50, 1.06]	< 0.00001	NA
	physical functions	SMD 0.14 [−0.10, 0.38]	0.25	0%
	self-care skils	SMD 0.15 [−0.47, 0.77]	0.63	79%
	life satisfaction	SMD 0.06 [−0.37, 0.48]	0.79	0%
	pooled results	SMD 0.11 [−0.10, 0.32]	0.31	76%
WHOQOL-OLD	death and dying	SMD −0.46 [−1.04, 0.11]	0.11	83%
	autonomy	SMD −1.45 [−3.49, 0.60]	0.17	98%
	past, present and future activities	SMD −1.48 [−2.44, − 0.52]	0.002	92%
	intimacy	SMD −1.15 [−1.88, − 0.43]	0.002	88%
	social participation	SMD −1.29 [−2.34, − 0.24]	0.02	94%
	sensory abilities	SMD −1.06 [−1.80, −0.33]	0.005	88%
	pooled results	SMD −1.13 [−1.47, −0.80]	< 0.00001	91%
*WHOQOL-BREF*	general health	SMD −0.24 [1.00, 0.52]	0.54	92%
	physical health	SMD −0.69 [−1.17, − 0.22]	0.004	91%
	psychological health	SMD −0.82 [−1.40, − 0.24]	0.006	94%
	social relationship	SMD −0.88 [− 1.46, − 0.29]	0.003	94%
	environmental area	SMD −0.66 [− 1.26, − 0.07]	0.03	94%
	pooled results	SMD −0.70 [− 0.94, − 0.47]	< 0.00001	93%
SD-36 and RAND-36	physical functioning	SMD −21.74 [−35.70, −7.79]	0.002	81%
	general health perceptions	SMD −2.06 [−6.31, 2.19]	0.34	5%
	role emotional	SMD −5.99 [−26.18, 14.20]	0.56	85%
	bodily pain	SMD 2.50 [−14.93, 19.92]	0.78	88%
	mental health	SMD −10.39 [−21.53, 0.75]	0.07	85%
	social functioning	SMD 4.35 [−8.21, 16.91]	0.5	92%
	role physical	SMD −12.30 [−46.79, 22.18]	0.48	94%
	vitality	SMD −4.52 [−12.36, 3.33]	0.26	74%
	pooled results	SMD −5.97 [−11.29, −0.64]	0.03	90%

Notes: *SMD* Standard Mean Difference, *NA* Not Applicable

**Table 6:** Evidence profile of quality of life of institutionalized and non-institutionalized elderly for LEIPAD questionnaire

Certainty assessment							Summary of findings			
№ of participants (studies) Follow-up	Risk of bias	Inconsistency	Indirectness	Imprecision	Publication bias	Overall certainty of evidence	Study event rates (%)		Anticipated absolute effects	
							With NIE	With IE	Risk with NIE	Risk difference with IE
LEIPAD – Overall										
1765 (2 observational studies)	very serious ^a^	very serious ^b,c^	not serious	not serious	very strong association all plausible residual confounding would suggest spurious effect, while no effect was observed	⨁◯◯◯ VERY LOW	1009	756	–	SMD 0.11 higher (0.1 lower to 0.32 higher)
LEIPAD - Physical functions										
280 (2 observational studies)	very serious ^a^	not serious	not serious	serious ^d^	very strong association all plausible residual confounding would suggest spurious effect, while no effect was observed	⨁⨁◯◯ LOW	161	119	–	SMD 0.14 higher (0.1 lower to 0.38 higher)
LEIPAD - Self-care skills										
280 (2 observational studies)	very serious ^a^	very serious ^b,c^	not serious	very serious ^d, e^	very strong association all plausible residual confounding would suggest spurious effect, while no effect was observed	⨁◯◯◯ VERY LOW	161	119	–	SMD 0.15 higher (0.47 lower to 0.77 higher)
LEIPAD - Cognitive functions										
280 (2 observational studies)	very serious ^a^	not serious	not serious	serious ^d^	strong association all plausible residual confounding would suggest spurious effect, while no effect was observed	⨁◯◯◯ VERY LOW	161	119	–	SMD 0.26 lower (0.5 lower to 0.02 lower)
LEIPAD - Depression and anxiety										
280 (2 observational studies)	very serious ^a^	not serious	not serious	serious ^d^	strong association all plausible residual confounding would suggest spurious effect, while no effect was observed	⨁◯◯◯ VERY LOW	161	119	–	SMD 0.24 lower (0.48 lower to 0)
LEIPAD - Social functions										
280 (2 observational studies)	very serious ^a^	not serious	not serious	serious ^d^	strong association all plausible residual confounding would suggest spurious effect, while no effect was observed	⨁◯◯◯VERY LOW	161	119	–	SMD 0.35 SD higher (0.11 higher to 0.59 higher)
LEIPAD - Sexual functions										
280 (2 observational studies)	very serious ^a^	not serious	not serious	serious ^d^	all plausible residual confounding would suggest spurious effect, while no effect was observed	⨁◯◯◯ VERY LOW	161	119	–	SMD 0.78 higher (0.5 higher to 1.06 higher)
LEIPAD - Life Satisfaction										
85 (2 observational studies)	very serious ^a^	not serious	not serious	serious ^d^	very strong association all plausible residual confounding would suggest spurious effect, while no effect was observed	⨁⨁◯◯ LOW	43	42	–	SMD 0.06 higher (0.37 lower to 0.48 higher)

Notes: *SMD* Standard mean difference; ^a^ Only studies with some risk of bias were included in this analysis; ^b^ Considerable heterogeneity; ^c^ There is wide variation in the effect estimates across studies with little or no overlap of confidence intervals associated with the effect estimates; ^d^ Total number of participants is less than 400; ^e^ Upper and lower confidence limit crosses the effect size were greater than 0.5

### WHOQOL-BREF questionnaire

**Fig. 3:**
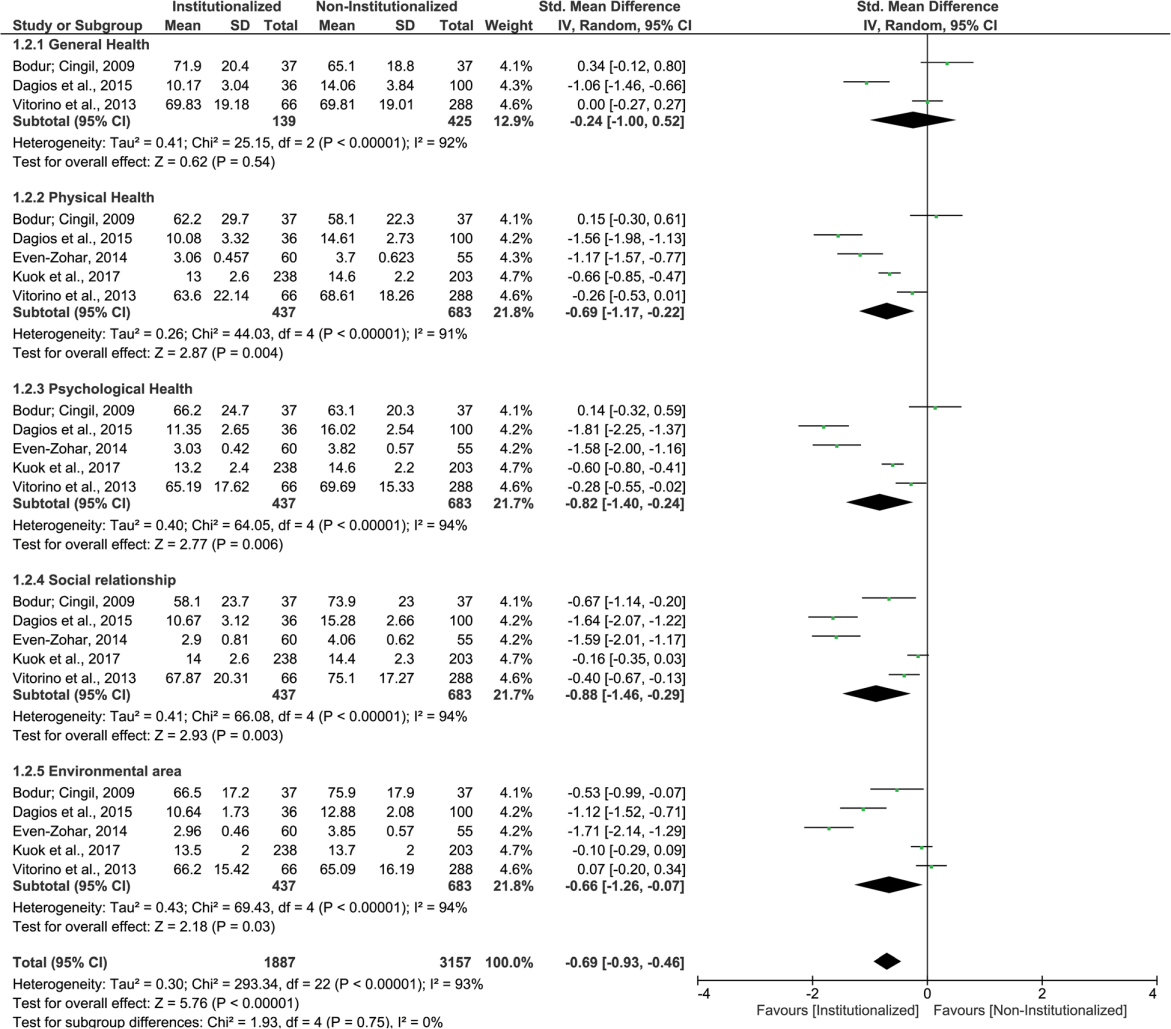
Forest plot of the influence of institutionalization on the elderly’s quality of life according to the studies that used WHOQOL-BREF questionnaire

Five studies were included in this second MA. Institutionalized elderly and non- institutionalized elderly presented similar mean scores (similar QoL) only for ‘general health’ domain, with very low certainty of evidence. For all other domains, as well as for pooled results, institutionalized elderly presented lower mean scores (worse QoL) than non- institutionalized elderly – ‘physical health’, ‘psychological health’, ‘social relationship’, ‘environmental area’, overall (Fig. 3 and Table 5). All domains were classified as having very low certainty of evidence, while overall result was classified with low certainty of evidence. Table 7 describes GRADE classifications and reasons for each WHOQOL-BREF questionnaire domain and pooled results.

**Table 7:** Evidence profile of quality of life of institutionalized and non-institutionalized elderly for WHOQOL-BREF questionnaire

Certainty assessment							Summary of findings			
№ of participants (studies) Follow-up	Risk of bias	Inconsistency	Indirectness	Imprecision	Publication bias	Overall certainty of evidence	Study event rates (%)		Anticipated absolute effects	
							With NIE	With IE	Risk with NIE	Risk difference with IE
WHOQOL-BREF – Overall										
5044 (5 observational studies)	not serious	serious ^a^	not serious	not serious	all plausible residual confounding would suggest spurious effect, while no effect was observed	⨁⨁◯◯LOW	3157	1887	–	SMD 0.69 lower(0.93 lower to 0.46 lower)
WHOQOL-BREF - General Health										
564 (3 observational studies)	very serious ^b^	very serious ^a,c^	not serious	serious ^d^	strong associationall plausible residual confounding would suggest spurious effect, while no effect was observed	⨁◯◯◯VERY LOW	425	139	–	SMD 0.24 lower(1.0 lower to 0.52 higher)
WHOQOL-BREF - Physical Health										
1120 (5 observational studies)	not serious	very serious ^a,c^	not serious	not serious	all plausible residual confounding would suggest spurious effect, while no effect was observed	⨁◯◯◯VERY LOW	683	437	–	SMD 0.69 lower(1.17 lower to 0.22 lower)
WHOQOL-BREF - Psychological Health										
1120 (5 observational studies)	not serious	very serious ^a,c^	not serious	serious ^d^	all plausible residual confounding would suggest spurious effect, while no effect was observed	⨁◯◯◯VERY LOW	683	437	–	SMD 0.82 lower(1.4 lower to 0.24 lower)
WHOQOL-BREF - Social relationship										
1120 (5 observational studies)	serious ^e^	serious ^a^	not serious	serious ^d^	all plausible residual confounding would suggest spurious effect, while no effect was observed	⨁◯◯◯VERY LOW	683	437	–	SMD 0.88 lower(1.46 lower to 0.29 lower)
WHOQOL-BREF - Environmental area										
1120 (5 observational studies)	serious ^f^	serious ^a^	not serious	serious ^d^	all plausible residual confounding would suggest spurious effect, while no effect was observed	⨁◯◯◯VERY LOW	683	437	–	SMD 0.66 lower(1.26 lower to 0.07 lower)

Notes: *SMD* Standard mean difference, ^a^ Considerable heterogeneity; ^b^ Only studies with some risk of bias were included in this analysis; ^c^ There is wide variation in the effect estimates across studies with little or no overlap of confidence intervals associated with the effect estimates; ^d^ Upper and lower confidence limit crosses the effect size were greater than 0.5; ^e^ Effect and significance (*p* value) change after exclusion of studies with risk of bias (SMD -0.16 [−0.35, 0.03] *p* = 0.09); f Effect and significance (*p* value) change after exclusion of studies with risk of bias (SMD -0.10 [− 0.29, 0.09] *p* = 0.3)

### WHOQOL-OLD questionnaire

**Fig. 4:**
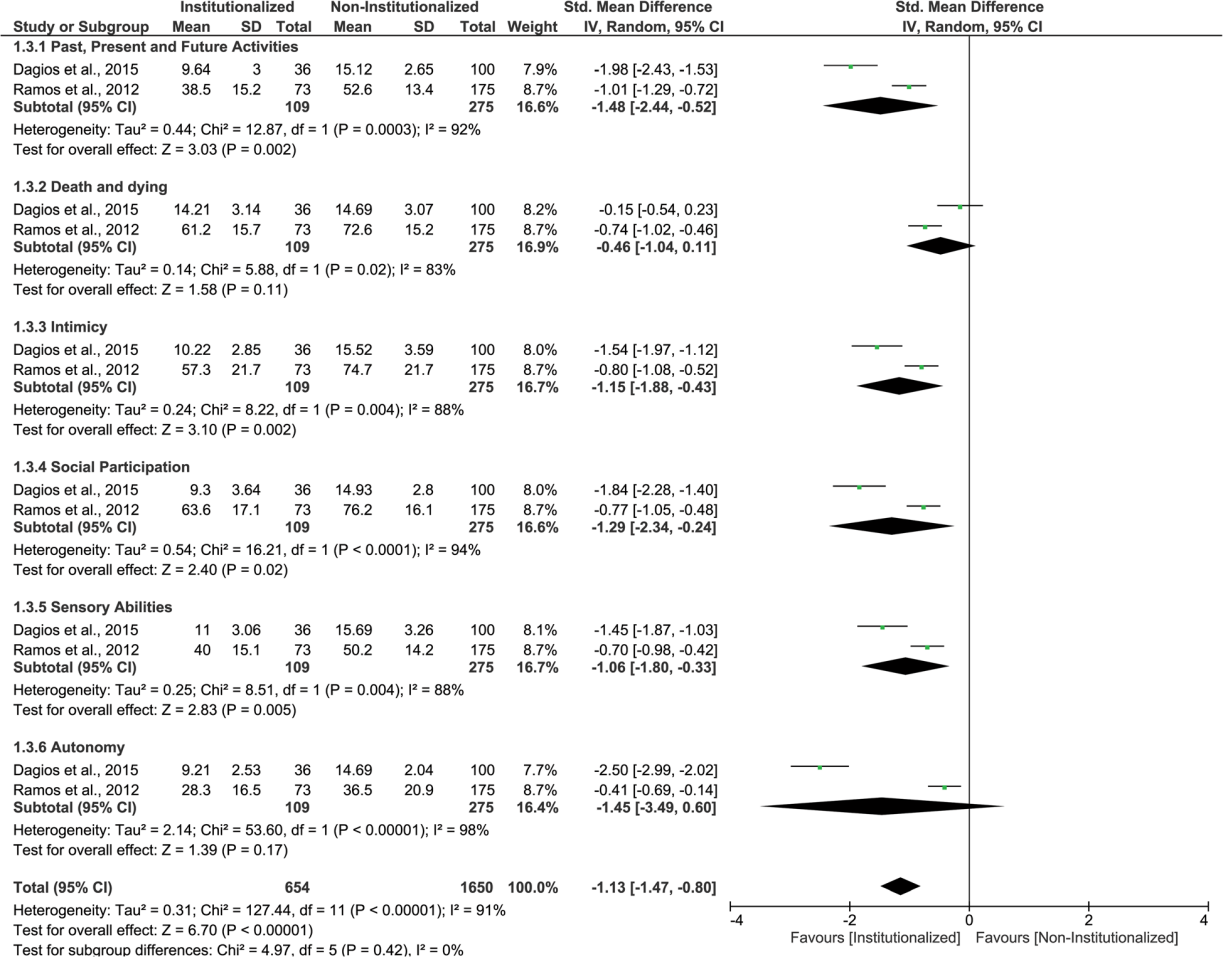
Forest plot of the influence of institutionalization on the elderly’s quality of life according to the studies that used WHOQOL-OLD questionnaire

Two studies were included in this third MA. institutionalized elderly and non- institutionalized elderly presented similar mean scores for ‘death and dying’ and ‘autonomy’ domains with very low certainty of evidence. However, for ‘past, present and future activities’, ‘intimacy’, ‘social participation’ and ‘sensory abilities’ domains, as well as for pooled results, institutionalized elderly presented lower mean scores (worse QoL) than non-institutionalized elderly (Fig. 4 and Table 5). All results were classified having low certainty of evidence. The GRADE classifications and reasons for each WHOQOL-OLD questionnaire domain and pooled results are in Table 8.

**Table 8:** Evidence profile of quality of life of institutionalized and non-institutionalized elderly for WHOQOL-OLD questionnaire

Certainty assessment							Summary of findings			
№ of participants (studies) Follow-up	Risk of bias	Inconsistency	Indirectness	Imprecision	Publication bias	Overall certainty of evidence	Study event rates (%)		Anticipated absolute effects	
							With NIE	With IE	Risk with NIE	Risk difference with IE
WHOQOL-OLD										
2304 (2 observational studies)	very serious ^a^	serious ^b^	not serious	serious ^c^	all plausible residual confounding would suggest spurious effect, while no effect was observed	⨁◯◯◯VERY LOW	1650	654	–	SMD 1.13 lower(1.47 lower to 0.8 lower)
WHOQOL-OLD - Past, Present and Future Activities										
384 (2 observational studies)	very serious ^a^	serious ^b^	not serious	very serious ^c, d^	all plausible residual confounding would suggest spurious effect, while no effect was observed	⨁◯◯◯VERY LOW	275	109	–	SMD 1.48 lower(2.44 lower to 0.52 lower)
WHOQOL-OLD - Death and dying										
384 (2 observational studies)	serious ^a^	serious ^b^	not serious	very serious ^c, d^	strong associationall plausible residual confounding would suggest spurious effect, while no effect was observed	⨁◯◯◯VERY LOW	275	109	–	SMD 0.46 lower(1.04 lower to 0.11 higher)
WHOQOL-OLD – Intimicy										
384 (2 observational studies)	very serious ^a^	serious ^b^	not serious	very serious ^c, d^	all plausible residual confounding would suggest spurious effect, while no effect was observed	⨁◯◯◯VERY LOW	275	109	–	SMD 1.15 lower(1.88 lower to 0.43 lower)
WHOQOL-OLD - Social Participation										
384 (2 observational studies)	very serious ^a^	serious ^b^	not serious	very serious ^c, d^	all plausible residual confounding would suggest spurious effect, while no effect was observed	⨁◯◯◯VERY LOW	275	109	-	SMD 1.29 lower(2.34 lower to 0.24 lower)
WHOQOL-OLD - Sensory Abilities										
384 (2 observational studies)	very serious ^a^	serious ^b^	not serious	very serious ^c, d^	all plausible residual confounding would suggest spurious effect, while no effect was observed	⨁◯◯◯VERY LOW	275	109	–	SMD 1.06 lower(1.8 lower to 0.33 lower)
WHOQOL-OLD – Autonomy										
384 (2 observational studies)	very serious ^a^	serious ^b^	not serious	very serious ^c, d^	all plausible residual confounding would suggest spurious effect, while no effect was observed	⨁◯◯◯VERY LOW	275	109	-	SMD 1.45 lower(3.49 lower to 0.6 higher)

Notes: *SMD* Standard mean difference, ^a^ Only studies with some risk of bias were included in this analysis; ^b^ Considerable heterogeneity; ^c^ Total number of participants is less than 400; ^d^ There is wide variation in the effect estimates across studies with little or no overlap of confidence intervals associated with the effect estimates

### SD-36 RAND-36 questionnaire

**Fig. 5:**
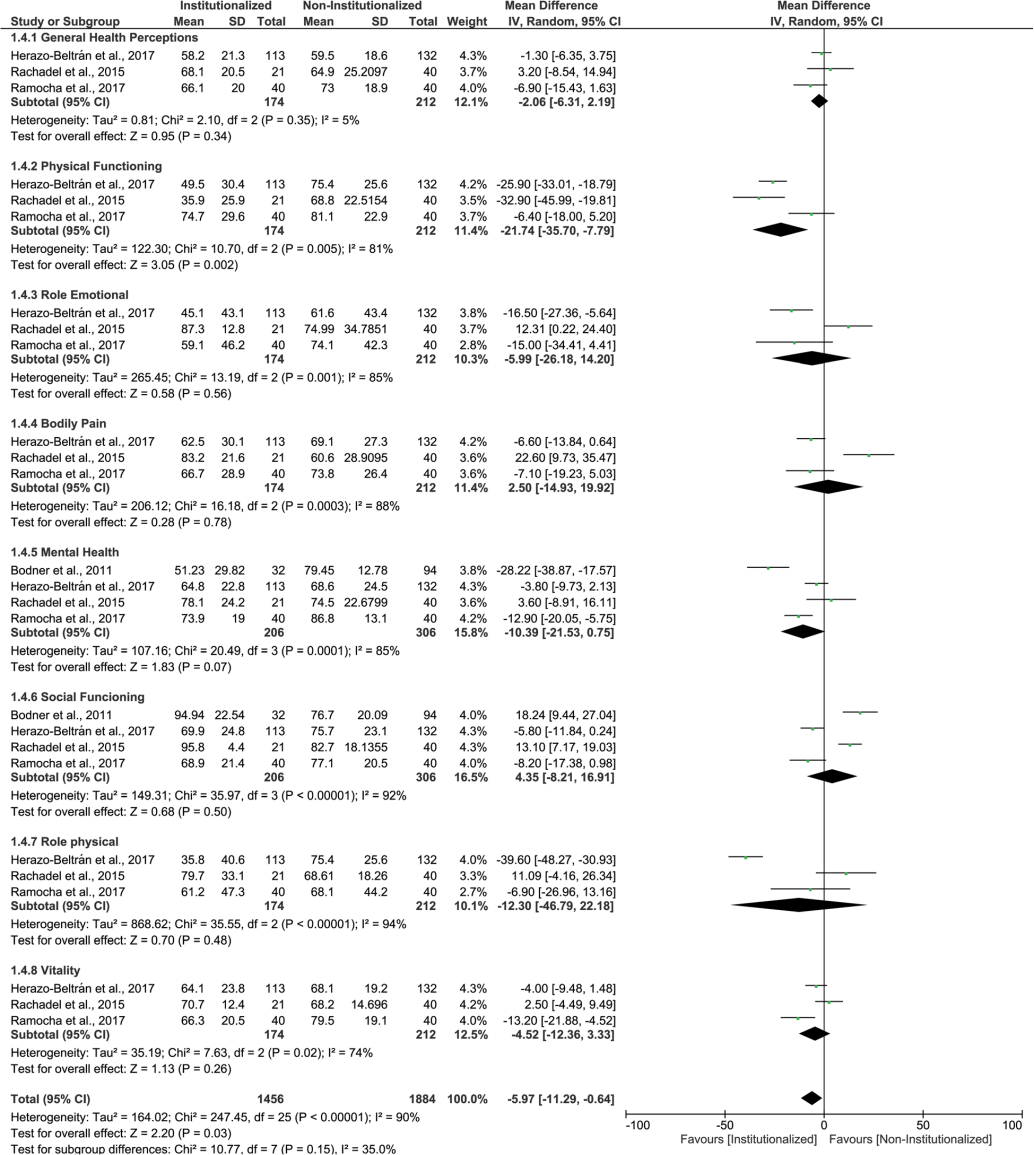
Forest plot of the influence of institutionalization on the elderly’s quality of life according to the studies that used SF-36 or RAND-36 questionnaire

Three studies were included in this fourth and last MA. The results indicate that institutionalized elderly presented lower mean scores (worse QoL) than non- institutionalized elderly for ‘physical functioning’ domain, as well as for pooled results. For all other domains, institutionalized elderly and non- institutionalized elderly presented similar mean scores (similar QoL) – ‘general health perceptions’, ‘role emotional’, ‘bodily pain’, ‘mental health’, ‘social functioning’, ‘role physical’, ‘vitality’ (Fig. 5 and Table 5). All results were classified having very low certainty of evidence. In Table 9, the GRADE classifications and reasons for each SF-36 and RAND-36 questionnaire domain and pooled results are described.

**Table 9:** Evidence profile of quality of life of institutionalized and non-institutionalized elderly for SD-36 and RAND-36 questionnaires

Certainty assessment							Summary of findings			
№ of participants (studies) Follow-up	Risk of bias	Inconsistency	Indirectness	Imprecision	Publication bias	Overall certainty of evidence	Study event rates (%)		Anticipated absolute effects	
							With NIE	With IE	Risk with NIE	Risk difference with IE
SF-36 and RAND-36 - Overall										
3340 (4 observational studies)	very serious ^a^	very serious ^b,c^	not serious	serious ^d^	very strong association	⨁◯◯◯VERY LOW	1884	1456	The mean SF-36 and RAND-36 was 0	MD 5.97 lower(11.29 lower to 0.64 lower)
SF-36 and RAND-36 - General Health Perceptions										
386 (3 observational studies)	very serious ^a^	not serious	not serious	very serious ^d, e^	strong association	⨁◯◯◯VERY LOW	212	174	The mean SF-36 and RAND-36 - General Health Perceptions was 0	MD 2.06 lower(6.31 lower to 2.19 higher)
SF-36 and RAND-36 - Physical Functioning										
386 (3 observational studies)	very serious ^a^	serious ^b^	not serious	very serious ^d, e^	very strong association	⨁◯◯◯VERY LOW	212	174	The mean SF-36 RAND-36 - Physical Functioning was 0	MD 21.74 lower(35.7 lower to 7.79 lower)
SF-36 and RAND-36 - Role Emotional										
386 (3 observational studies)	very serious ^a^	very serious ^b,c^	not serious	very serious ^d, e^	very strong association	⨁◯◯◯VERY LOW	212	174	The mean SF-36 RAND-36 - Role Emotional was 0	MD 5.99 lower(26.18 lower to 14.2 higher)
SF-36 and RAND-36 - Bodily Pain										
386 (3 observational studies)	very serious ^a^	very serious ^b,c^	not serious	very serious ^d, e^	strong association	⨁◯◯◯VERY LOW	212	174	The mean SF-36 RAND-36 - Bodily Pain was 0	MD 2.5 higher(14.93 lower to 19.92 higher)
SF-36 and RAND-36 - Mental Health										
512 (4 observational studies)	very serious ^a^	serious ^b^	not serious	serious ^d^	very strong association	⨁◯◯◯VERY LOW	306	206	The mean SF-36 RAND-36 (QVRS) - Mental Health was 0	MD 10.39 lower(21.53 lower to 0.75 higher)
SF-36 and RAND-36 - Social Functioning										
512 (4 observational studies)	very serious ^a^	very serious ^b,c^	not serious	serious ^d^	strong association	⨁◯◯◯VERY LOW	306	206	The mean SF-36 RAND-36 (QVRS) - Social Functioning was 0	MD 4.35 higher(8.21 lower to 16.91 higher)
SF-36 and RAND-36 - Role physical										
386 (3 observational studies)	very serious ^a^	very serious ^b,c^	not serious	very serious ^d, e^	very strong association	⨁◯◯◯VERY LOW	212	174	The mean SF-36 RAND-36 (QVRS) - Role physical was 0	MD 12.3 lower(46.79 lower to 22.18 higher)
SF-36 and RAND-36 - Vitality										
386 (3 observational studies)	very serious ^a^	serious ^b^	not serious	very serious ^d, e^	strong association	⨁◯◯◯VERY LOW	212	174	The mean SF-36 RAND-36 - Vitality was 0	MD 4.52 lower(12.36 lower to 3.33 higher)

Notes: *MD* Mean difference; ^a^ Only studies with some risk of bias were included in this analysis. ^b^ Considerable heterogeneity. ^c^ There is wide variation in the effect estimates across studies with little or no overlap of confidence intervals associated with the effect estimates. ^d^ Upper and lower confidence limit crosses the effect size were greater than 0.5. ^e^ Total number of participants is less than 400

## Discussion

The process of population aging is a global phenomenon that must be accompanied by the physical, psychological, social, economic and spiritual well-being of the elderly [6]. As a result of this aging process and the unavailability of family members to care for the elderly, the institutionalization of these individuals has increased [7]. In this sense, the homes for the aged should be able to provide good quality of life for their residents [9]. In contrast, this systematic review summarized that the institutionalization affects the QoL of elderly individuals.

In our systematic review, of 16 studies included, 15 [10–23, 25] were conducted in developing countries, and of these studies, seven were performed in Brazil [11, 14–16, 19, 21, 25]. In developed nations, the need for nursing homes is reduced due to the care given to the elderly by the State and the family, as well as the high purchasing power of the population that allows the elderly to remain in their homes receiving the health care they need [7, 8]. Moreover, in these countries the institutionalization of the elderly is related to the presence of specific health conditions such as dementia, Alzheimer’s disease or cognitive disorders [7]. On the other hand, in the developing countries there is a high rate of institutionalization of the elderly due to cultural, economic and family factors [8]. In this context, identifying differences in QoL of institutionalized older people compared to non-institutionalized ones has been shown to be of interest in studies in developing countries, especially in Brazil.

In the same way, most of the study participants were elderly with 60 years old or more, which is in accordance with the definition by World Health Organization (WHO) and United Nations. Inconsistently, two studies were against this classification [14, 20]. Kuok et al. (2017) and Bonan et al. (2008) included a cut-off level of 50 and 55 years old, respectively. The first study [20] selected 451 participants, of which 248, were residents of nursing homes with a mean age of 78.4 (+/− 8.3) years old, and the other 203 were community dwelling elderly, aged 64.1 (+/− 6.8) years old. The latter research [14] included elderly aged 70.3 (+/− 10.2 years). Both studies revealed that a small number of participants had less than 60 years and those were not institutionalized [14, 20]. Moreover, no differences have been observed on QoL of elderly from long-term care institutions when compared to community dwelling ones [14, 20], reaffirming that ages < 60 years did not compromise their results. Therefore, whereas the sample size of Kuok et al. (2017) and Bonan et al. (2008) was uniquely included in our qualitative assessments (not included in meta-analysis), both studies were kept in this systematic review, not impairing the results.

The effects of aging process with regards to general health perceptions, physical, psychological social and environment domains can be verified by means of QoL questionnaires [6]. Although it is considered a subjective and complex evaluation, the QoL has been extensively studied among elderly, once the perception of life changes during aging process and is influenced by individual’s perspectives about life and society [33]. Therefore, some questionnaires have been used to assess HRQoL, as example of Leipad, WHOQOL-BREF, WHOQOL-OLD, SF-36, RAND-36, and OHRQoL as GOHAI.

The Leipad questionnaire comprises of 49 self-assessed items grouped in seven core domains: self-care, physical, cognitive, social and sexual functions, depression and anxiety and life satisfaction [34]. Two eligible studies [10, 24] were submitted to a meta-analysis and identified better QoL in institutionalized elderly, when compared to the non-institutionalized elderly, in the “cognitive functions” and “depression and anxiety”. It can be hypothesized that institutionalized elderly accepts and get used to an institutionalized life along time [24]. Since there is an increase on social interaction, communicative activities, and performance of cognitive exercises, depression and anxiety symptoms drastically decrease [10]. All these factors contribute to maintain elderly’s cognitive function, which improves QoL [10, 24].

Indeed, when Leipad domains were analysed together, no differences have been found on the QoL of the institutionalized elderly compared to the non-institutionalized. This result may be attributed to the low power of certainty of scientific evidence of the studies [10, 24] due to the incompatibility between groups in relation to age, gender, socioeconomic conditions and comorbidities, the non-reduction of these characteristic discrepancies on statistical analysis [10] and insufficient sample size [24].

The WHOQOL questionnaire is an international recognized instrument from WHO to evaluate QoL. Besides the extended version (WHOQOL-100) [35], there is an abbreviated (WHOQOL-BREF) [36] and a specific version to evaluate elderly’s QoL (WHOQOL-OLD) [37]. The WHOQOL-BREF contains 26 items grouped in four domains: physical, psychological, environmental and social [36], while WHOQOL-OLD comprises of 24 items subdivided into 6 domains: sensorial ability, autonomy, past, present and future activities, social participation, death and dead, intimacy [37].

Regarding meta-analysis using WHOQOL-BREF questionnaire [12, 15, 17, 20, 25], institutionalized elderly presented worse QoL in all domains as well as in the pooled results when compared to the non-institutionalized group. In relation to the physical domain, the differences can be explained by the insufficient promotion of physical activities between elderly in long-term care institutions, or their lack of engagement on social activities, aggravated by serious systemic diseases [13, 15, 20]. These individual health conditions aggravate the sedentary lifestyle, compromising the elderly functional capacity and physical health [20, 25]. Also, the absence of physical activity can lead to the development of depressive symptoms, explaining the worse QoL found in psychological domain for institutionalized elderly when compared to community dwelling ones [20, 25].

Depression is a prevalent disease in institutionalized elderly and a predictor of a worse QoL in social domain [20]. In addition, the physical distance between elderly and family, relatives and friends impair their social life and, consequently, their perception about QoL on the social domain [13, 16, 17], exposing the worse QoL found in institutionalized elderly. Nevertheless, elderly are constantly sheltered against their own desire and do not receive family visits, contributing to the isolation [16, 25]. Another important aspect that compromises QoL on social domain of institutionalized elderly is the lack of opportunity to accomplish leisure activities, which impacts on social environment and social contact between these individuals [25].

In addition, the absence of socialization is directly related to the deterioration of physical and mental health of institutionalized elderly, accounting for the worse QoL on physical and psychological domains when compared to the community dwelling [25]. Finally, differences on environmental domain describes the negative feeling of elderly concerning the distance from their home, and the difficult to adapt to the new and unfamiliar place of residence [13].

Still, the differences on QoL found between institutionalized and community dwelling elderly must be observed with caution due to the risk of bias and the low certainty of evidence of included studies. Bodur and Cingil (2009), Dagios et al. (2015) and Vitorino et al. (2013) did not paired the age between groups, then institutionalized elderly were older than the non-institutionalized group. However, there is a relation between age increment and declined QoL of elderly on psychological, social and environmental domains [13]. Therefore, the discrepancy of age in that studies [13, 16, 25] may have affected the meta-analysis results.

In addition, WHOQOL-BREF should be used simultaneously with the WHOQOL-OLD when the QoL of elderly people is being evaluated to improve the data collection and get more precise results. Despite this, only one article [16] have adopted both, whereas some authors [13, 15, 17, 20, 25] preferred to apply one of the versions, perhaps as a way to shorten the data collection. Moreover, although the use of WHO questionnaires requires attention to fill all items correctly, of the studies that used WHOQOL-BREF questionnaire, two [17, 25] applied this instrument by more than one interviewer, which could have under or overestimate the answers, which may account to the risk of biased results.

A meta-analysis of the studies that used WHOQOL-OLD questionnaire [16, 23] demonstrated on “past, present and future activities”, “intimacy”, “social participation” and “sensory abilities” domains, as well as for pooled results, worse QoL for the institutionalized elderly than for the non-institutionalized ones. The term “past, present and future activities” refers to the satisfaction with the future, the desired opportunities, and recognition with what has been achieved throughout life [37]. It is known that the great majority of the elderly are not freely institutionalized of their own, but rather by family decision [16]. This finding suggests that the elderly consider that being institutionalized is not what they hoped to have achieved in life, and that there are no opportunities to change this reality [16].

The ‘intimacy’ domain included questions about the sense of fellowship and love in life, and as opportunities to love and be loved. By any means, there is a prevalence of widowed, separated or single institutionalized elderly, that is, they do not have a partner, unlike the community dwelling elderly who are mostly married [17]. This explains the finding that the institutionalized elderly felt less satisfied about the companionship and love received than the non-institutionalized elderly. The satisfaction with the use of time, activity accomplishment and participation in the community are evaluated in the ‘social participation’ facet [37]. Thus, elderly residing in nursing homes cause a feeling of being prevented from carrying out their projects. This way, distance from family and friends also affects the social relations of these elderly, compromising their QoL in the ‘social participation’ domain [16].

At least, “sensory abilities” domain refers to the loss of sensory functioning in everyday life and in the ability to interact. In this context, institutionalized older people are more physically and sensorially incapacitated than the elderly living in the community [16, 23], confirming the results. Yet, these results might be interpreted with care, since the studies included in the meta-analysis refers to WHOQOL-OLD questionnaire [16, 23] presented methodological major problems that resulted in low certainty of scientific evidence.

The SF-36 and RAND-36 questionnaires comprises of 36 questions grouped in eight domains: physical functioning, role physical, bodily pain, general health perceptions, role emotional, vitality, mental health and social functioning [38, 49]. Although the SF-36 and Rand-36 instrument were considered a short form tool for health survey, both questionnaires represent a set of generic, coherent, and easily administered quality-of-life measurements [38, 39]. Furthermore, these instruments were used for several studies to assess health related QoL [18, 22, 23].

Considering the equality of the domains and the overall scale of these questionnaires and that they only differ slightly in the scoring method [39], the studies [18, 22, 23] that used SF-36 and RAND-36 questionnaires to evaluated the QoL were grouped in the same meta-analysis, in order to the quantitative synthesis was able to be performed. This meta-analysis demonstrated that the institutionalized elderly presented worse QoL than non-institutionalized elderly for “physical functioning” domain and pooled results. It brings out that the raised prevalence of health problems, such as degenerative joint disease [13], especially in institutionalized elderly limit the performance of physical activities.

However, SF-36 “physical functioning” domain evaluates the performance of vigorous activities that elderly generally cannot execute [40]. Possibly, questions within this domain do not measure accurately the performance of elderly with a poor systemic health [40], which is the case of the institutionalized elderly. All factors may have influenced the results found in the studies using SF-36 questionnaire [18, 22]. In addition, the study of Rachadel et al. (2015) the elderly who lived in nursing homes presented higher mean age than the non-institutionalized. This methodologic problem may affect the findings since the aging process is related to worse QoL of elderly [13].

Apart from the previous findings, the comparation of the OHRQoL between institutionalized elderly and community-dwelling elderly has not been frequently evaluated in the studies. Instead, only a few reports [11, 14] evaluated OHRQoL and revealed sparse results for institutionalized elderly when compared to the non-institutionalized elderly. Due to insufficient data, the meta-analysis did not include these two studies. Even though, the relevance of oral health conditions must be enhanced in further analysis, as the presence of teeth or prosthetic treatment improves self-steam and increase masticatory functions and, consequently, the elderly QoL [41–43].

Finally, it is essential to mention the limitations of the present systematic review, especially those concerning the different methodological measurements, the wide range of age, culture and gender found in the included studies. These limitations could be the main reason of the high heterogeneity [28, 31]. Therefore, our outcomes should be carefully observed, as it may not impact the elderly’s QoL worldwide. However, although the included studies used different questionnaires to assess the QoL, separate meta-analyzes were performed for each questionnaire [30] and the standardized mean difference was used when the studies measure the QoL in different scales [31]. These procedures were realized in order to minimize risk of bias and try to ensure the accuracy of the results.

The wide range of age could be explained due to most of the included studies were conducted in underdeveloped countries, where the mean age of elderly is lower than in developed countries [3] and the nursing homes do not have an age limit to admit people. Furthermore, the discrepancies of age, gender and culture is inherent to where and how the studies were conducted. Another limitation is the publication bias, which is the tendency of journals to publish positive results over negative evidence [28]. Thus, positive results of institutionalization over elderly’s QoL could have been found but never published before, which may bias the outcomes of this systematic review. In order to minimize this bias, we tried to identify unpublished works in SIGLE, in meetings and through contact with experts [28].

Nevertheless, although well-designed primary studies should be conducted to generate robust scientific studies to support the meta-analysis, no other review has been compiled data concerning QoL of institutionalized elderly and non-institutionalized elderly in the literature. Therefore, the outcomes of this study will help on guiding the creation of specific public health policies to the nursing homes. Regarding the low QoL found for institutionalized elderly, it is important to mention that health care must be provided and integrated to social services to ensure that dependent people keep the highest possible QoL [4]. Specialized professionals can be hired to work in nursing homes, according to the needs of each place, such as physical educators, physiotherapists, nutritionists, dentists, psychologists and medical doctors. Moreover, improving caregivers training and the infrastructure conditions guarantee QoL to the residents in physical, psychological, social and environmental aspects, and create an integrated environment where elderly could live with fairness, dignity, participation, respect and autonomy [9].

## Conclusion

The institutionalization influences negatively the QoL of the elderly. However, this should be approached with caution, due to the presence of methodological bias in the articles assessed in this systematic review, which consequently resulted in poor quality of evidence. Therefore, further primary and well delineated studies should be accomplished to confirm this evidence.

## Data Availability

The datasets used and/or analysed during the current study are available from the corresponding author on reasonable request. All data generated or analysed during this study are included in this published article and its supplementary information files.

## Abbreviations

CI: Confidence Interval
GRADE: Grading of Recommendations Assessment, Development and Evaluation
HRQoL: Health-Related Quality of Life
MA: Meta-Analysis
OHRQoL: Oral Health–Related Quality of Life
PECO: Population, Exposure, Comparison and Outcomes
PRISMA: Preferred Reporting Items for Systematic Reviews and Meta-Analysis
QoL: Quality of Life
SIGLE: System for Information on Gray Literature in Europe
VAS: Visual Analogue Scale
WHO: World Health Organization
